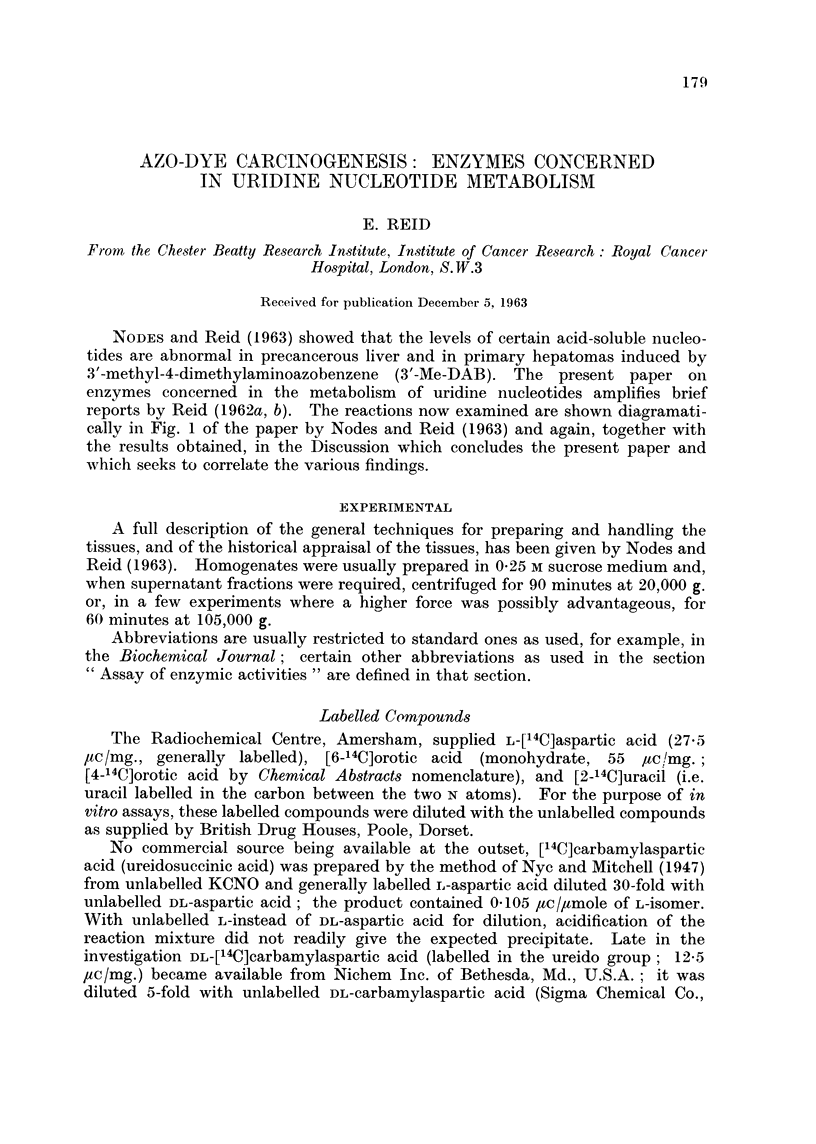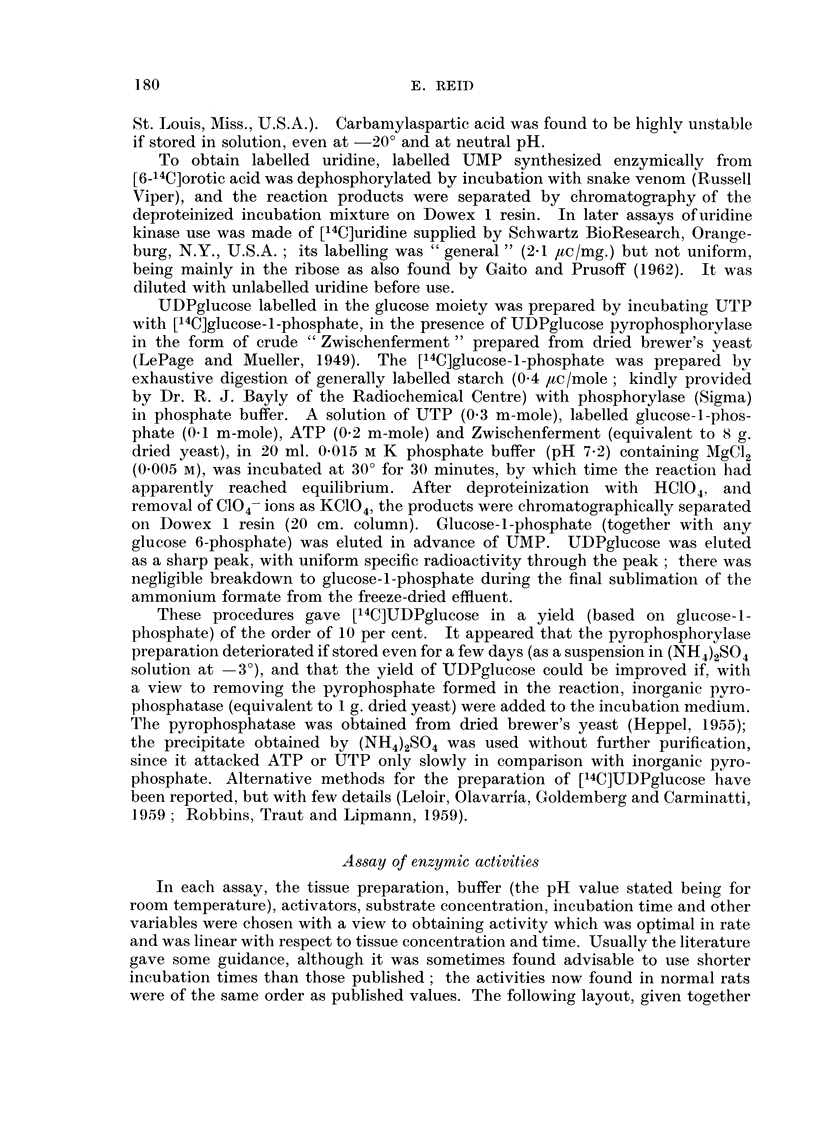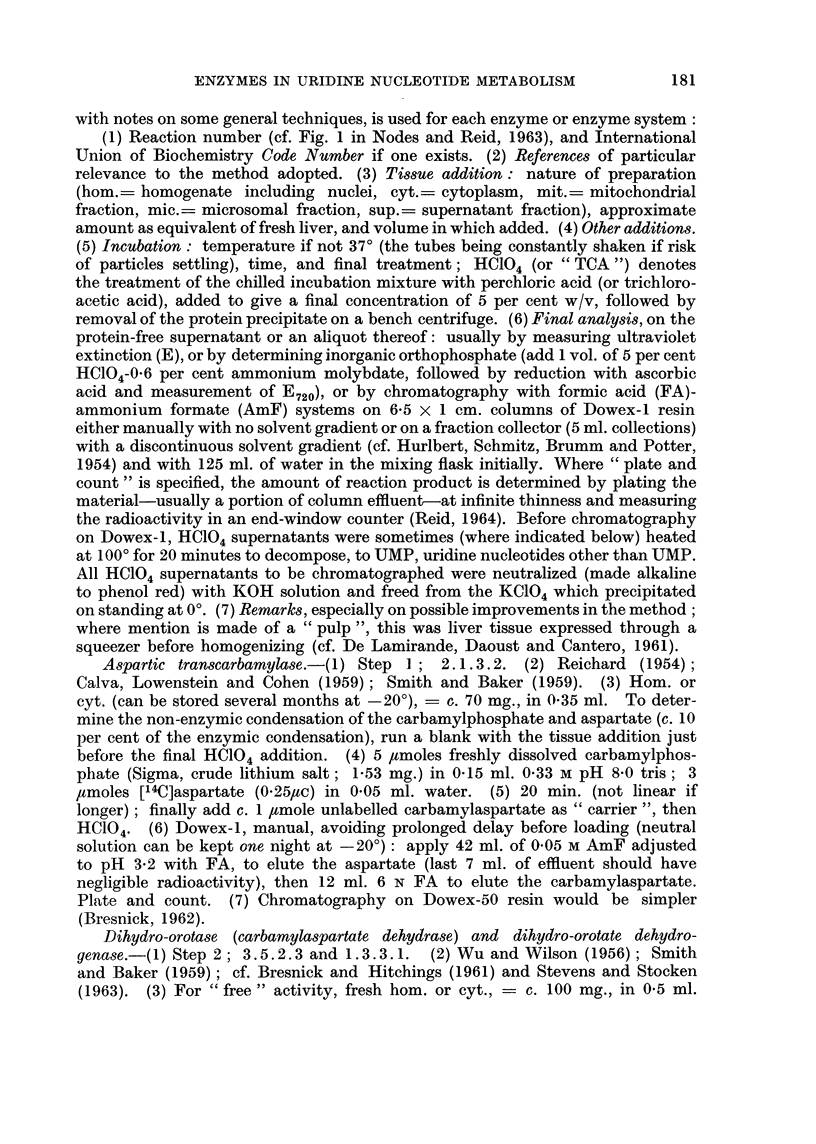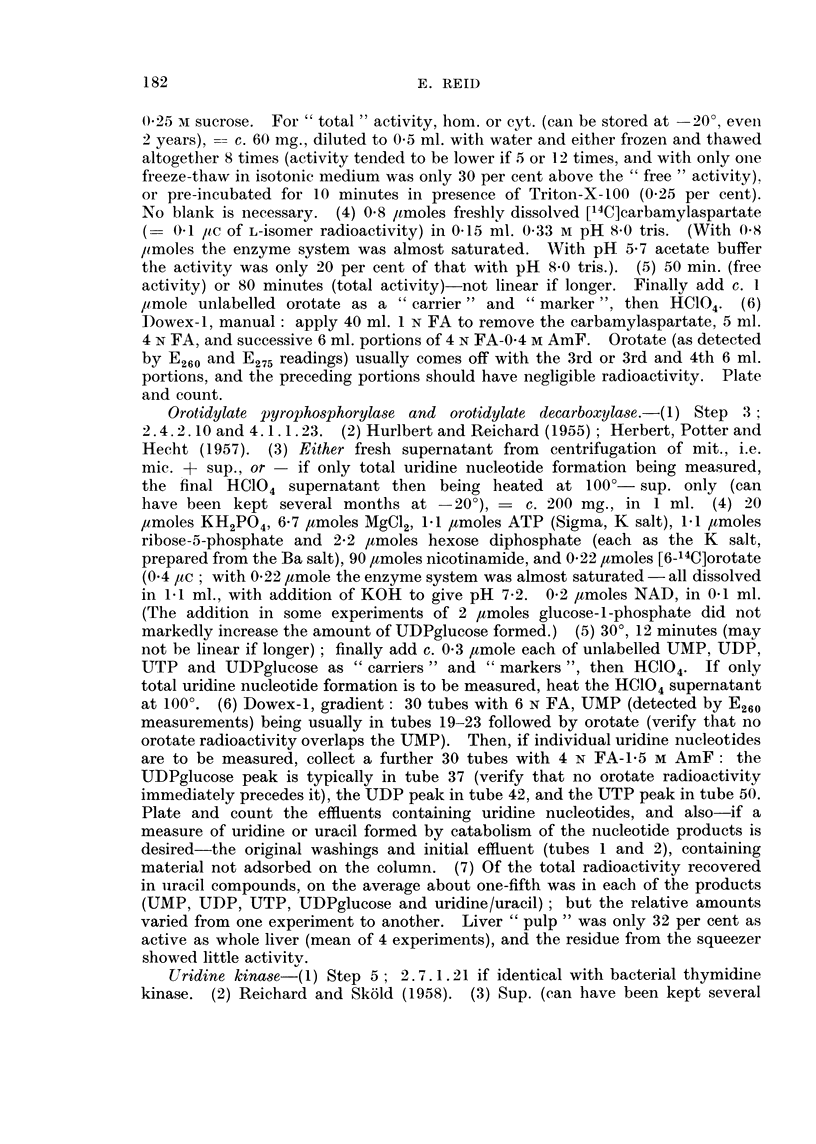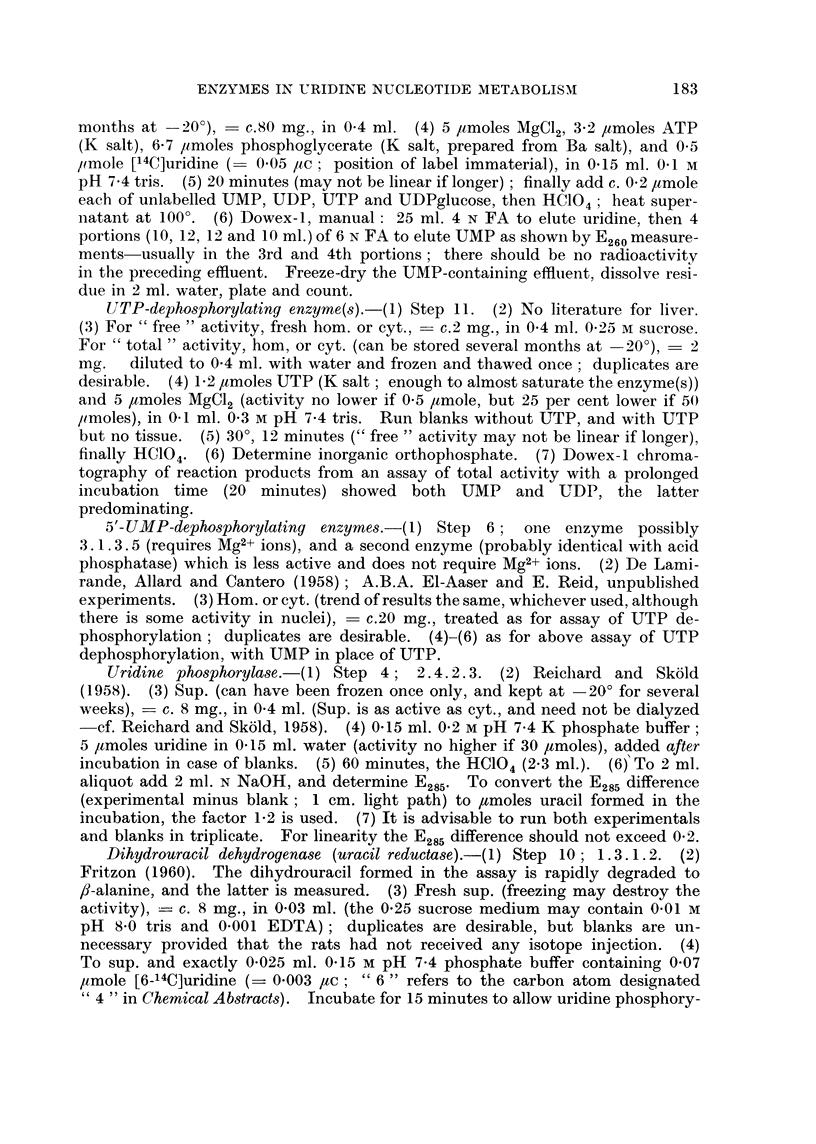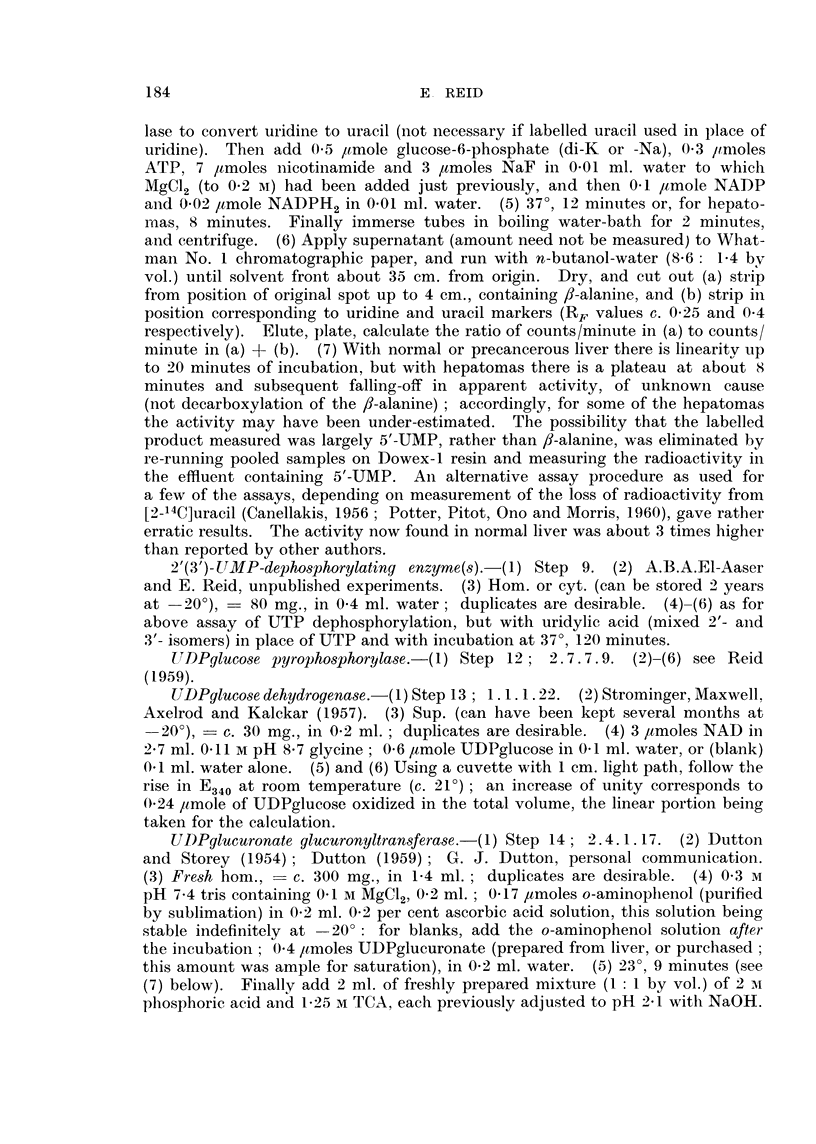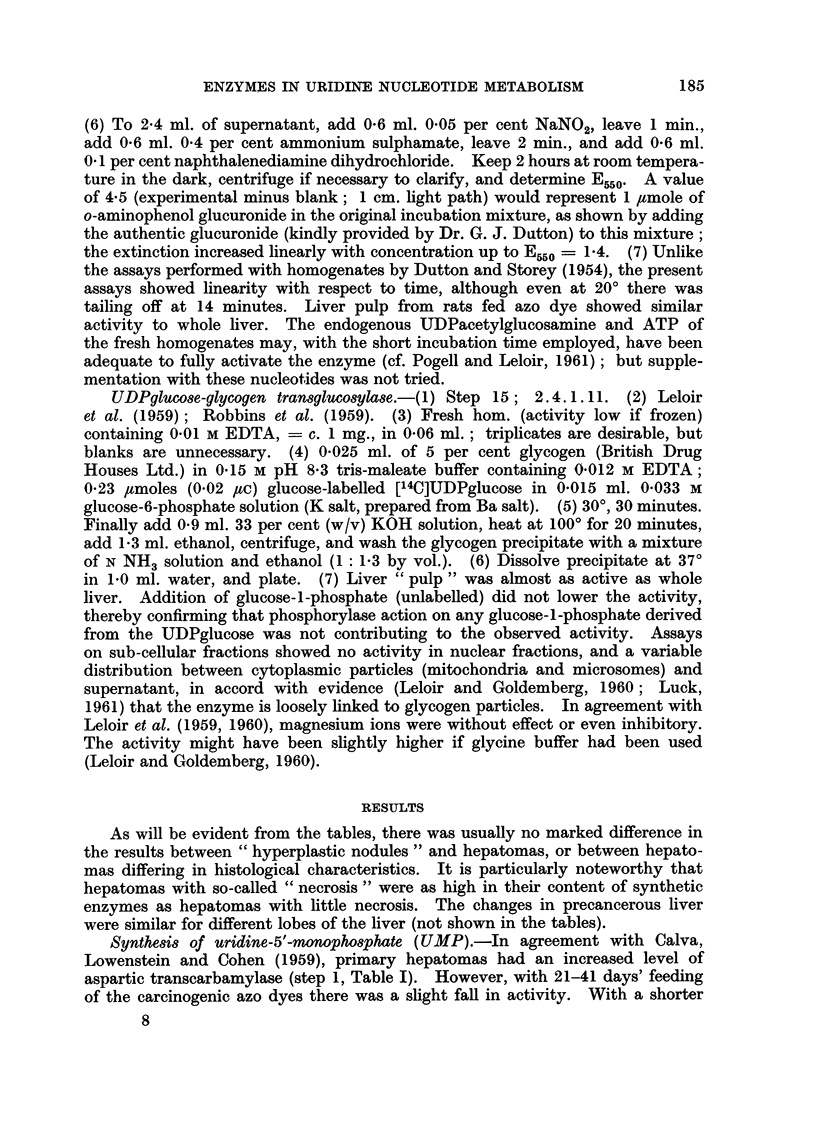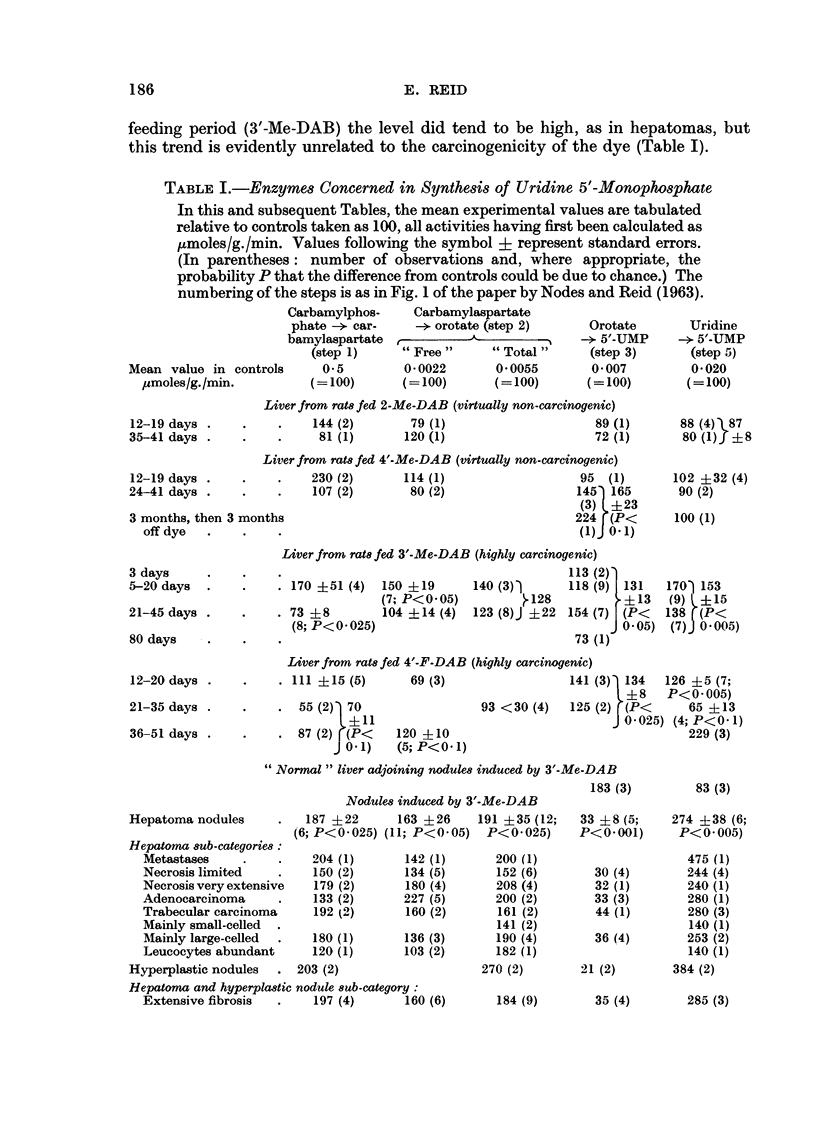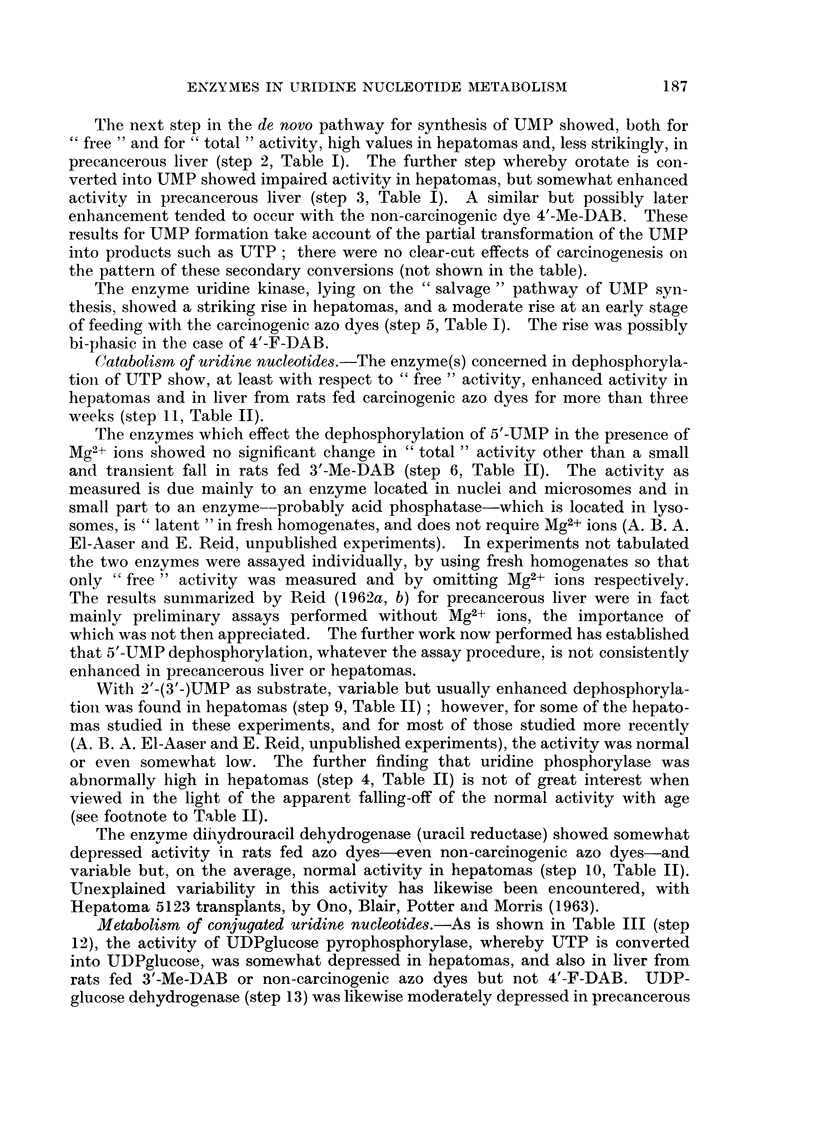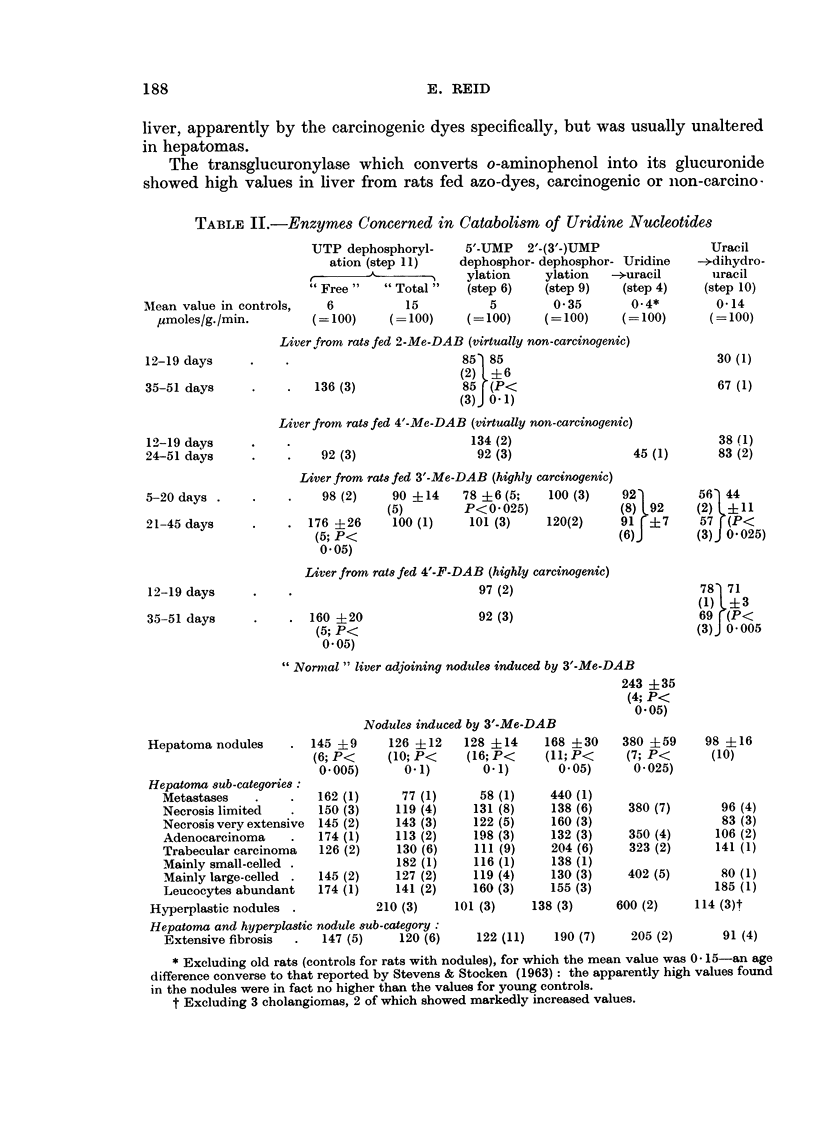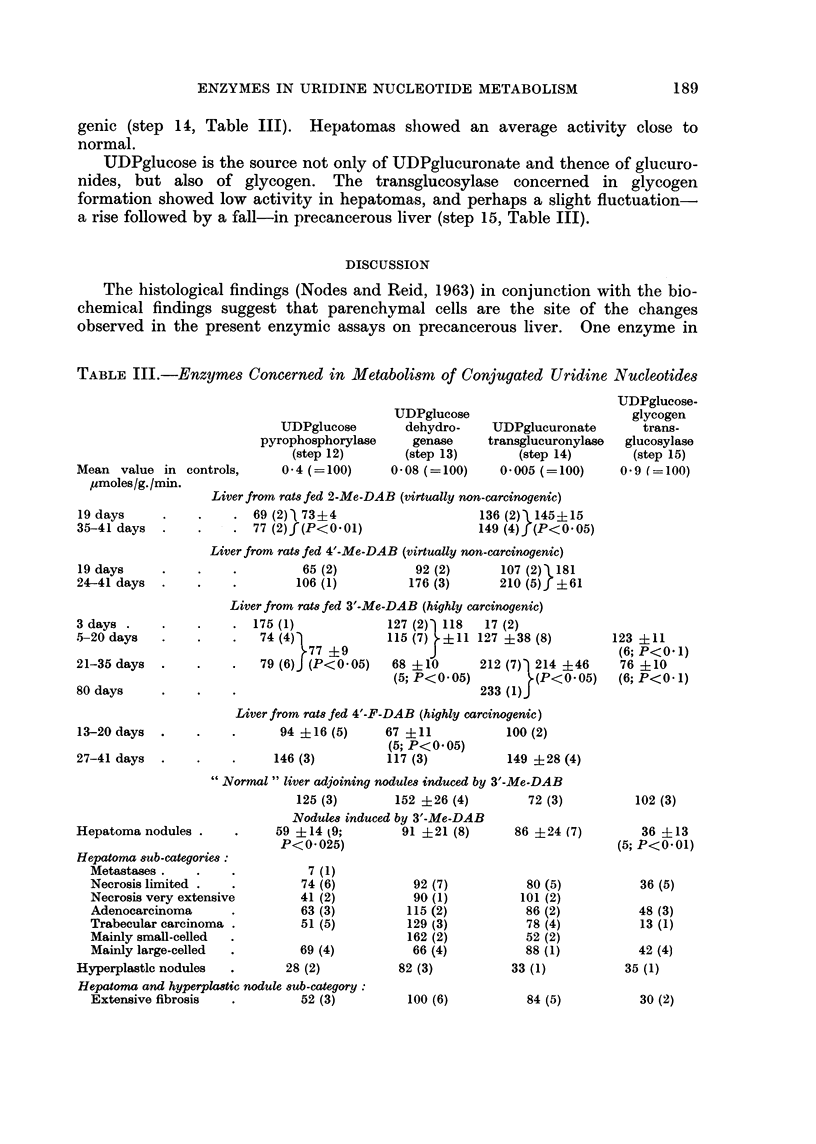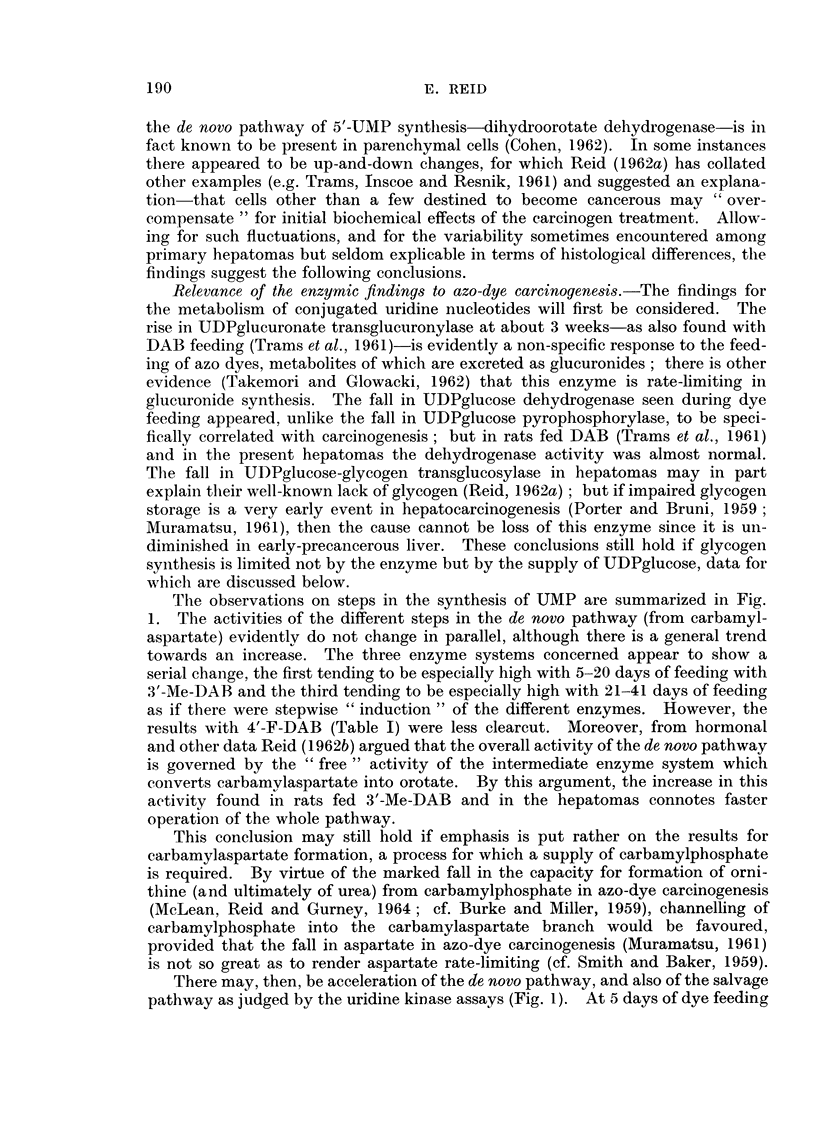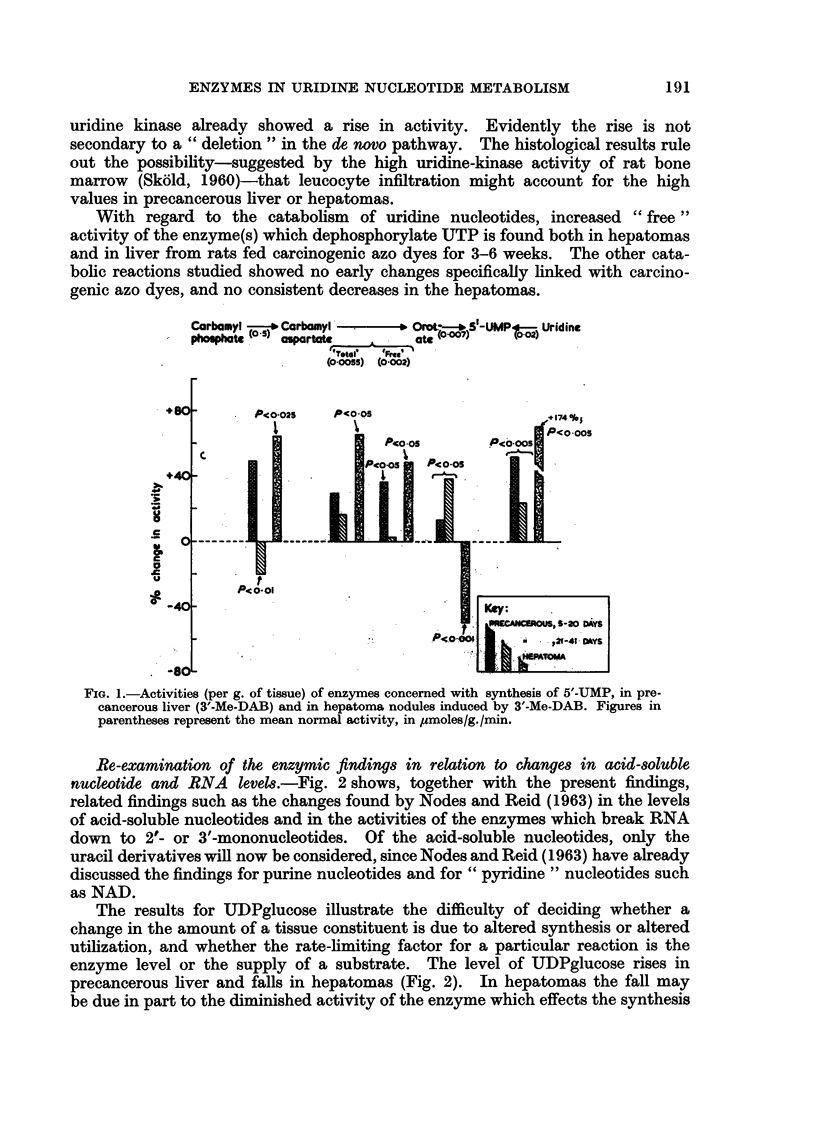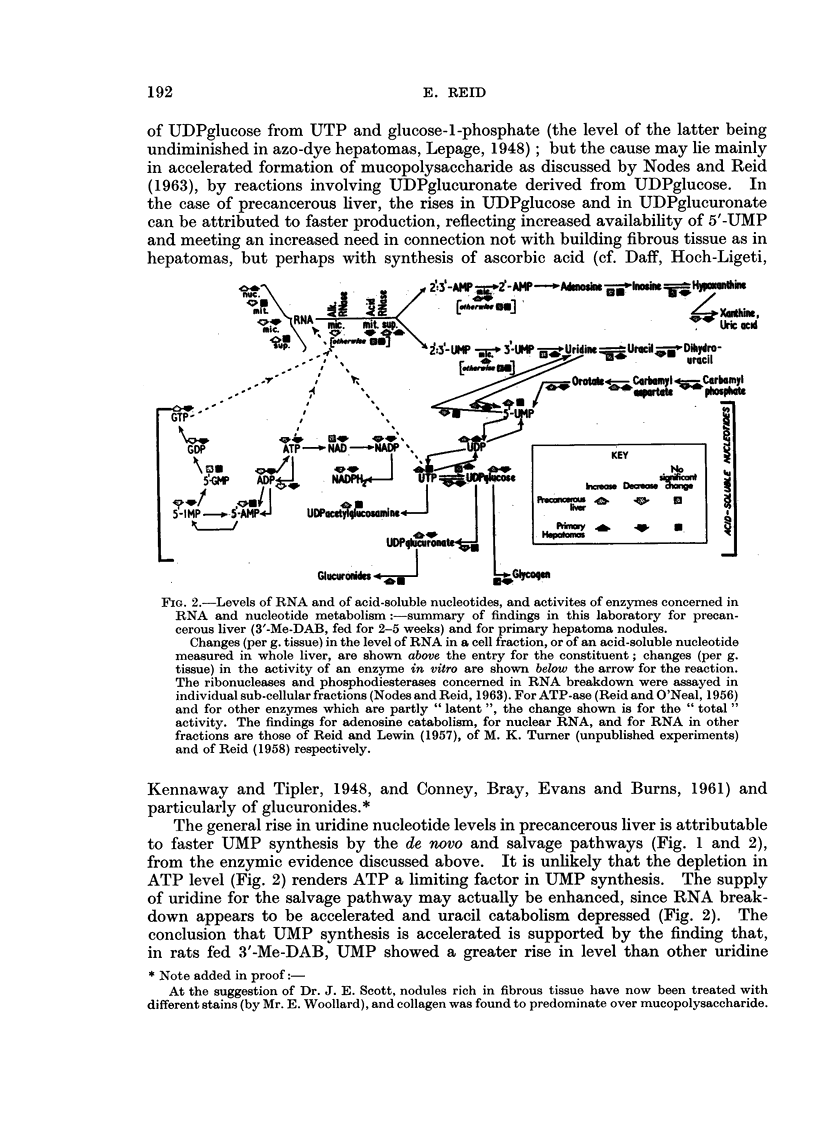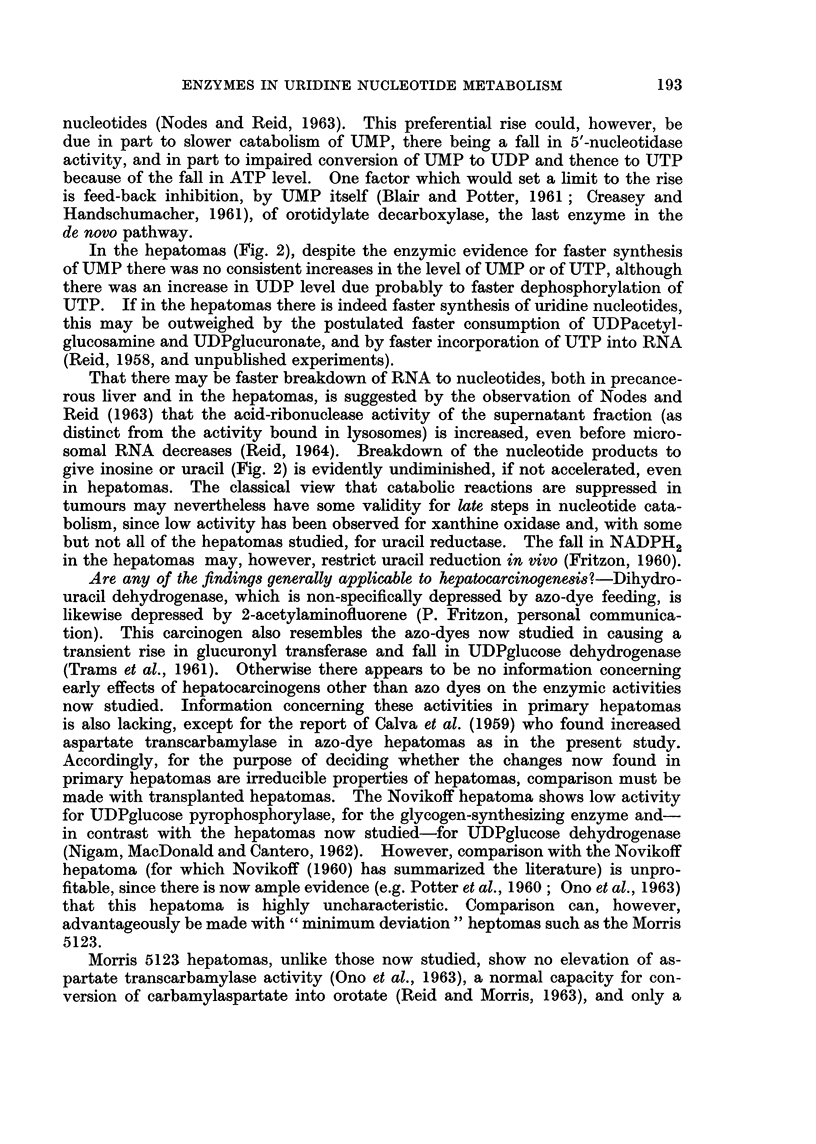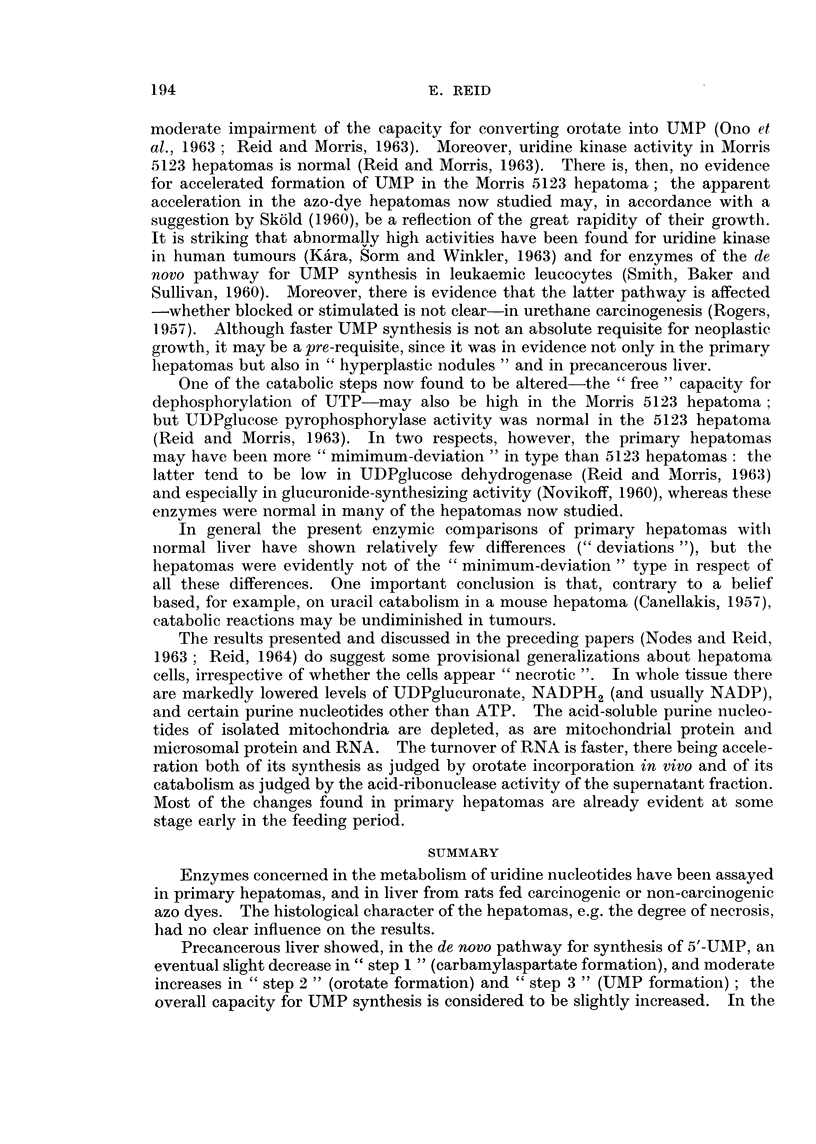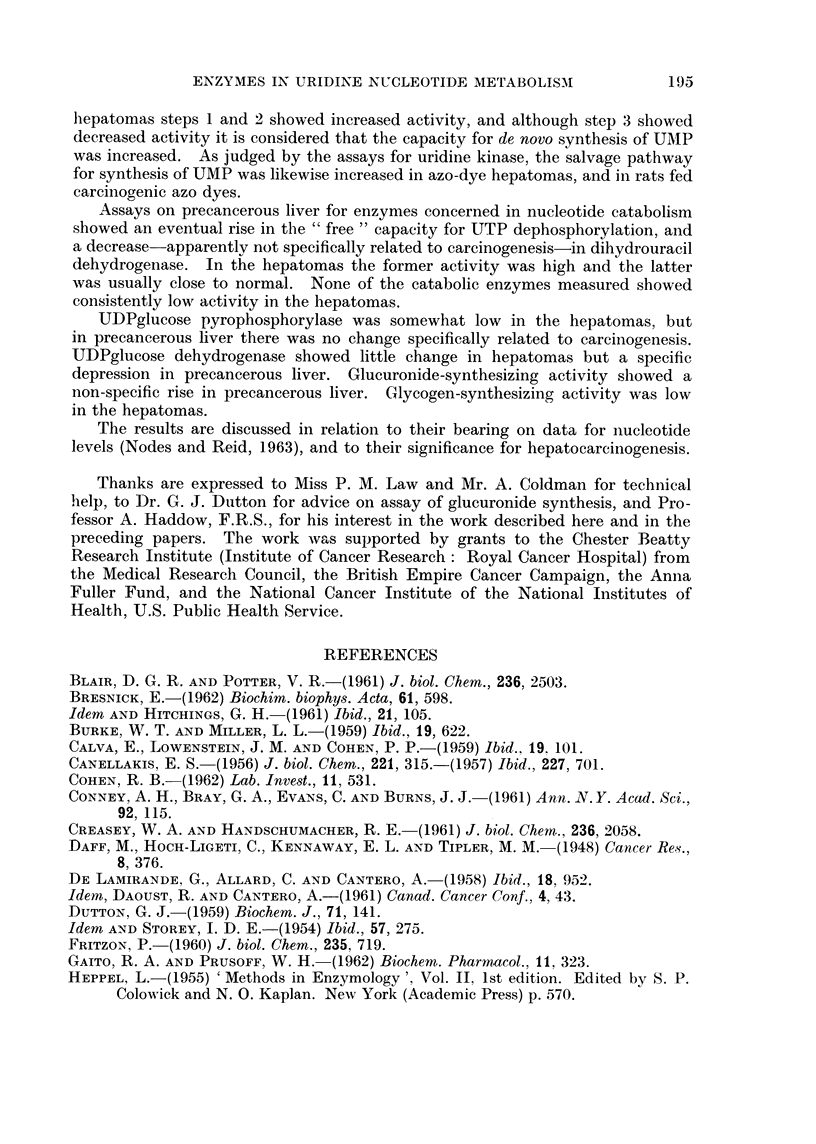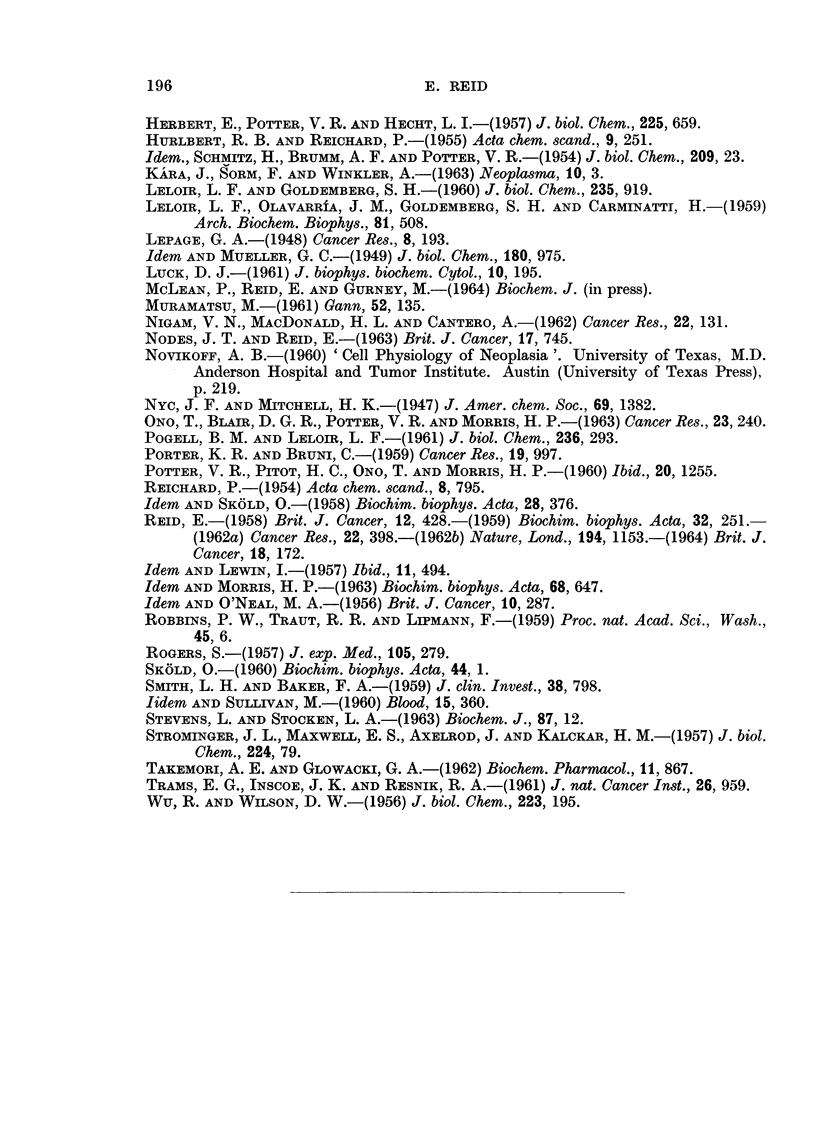# Azo-Dye Carcinogenesis: Enzymes Concerned in Uridine Nucleotide Metabolism

**DOI:** 10.1038/bjc.1964.21

**Published:** 1964-03

**Authors:** E. Reid


### AZO-DYE CARCINOGENESIS: ENZYMES CONCERNED IN URIDINE NUCLEOTIDE METABOLISM

E. REID

*From the Chester Beatty Research Institute, Institute of Cancer Research: Royal Cancer Hospital, London, S.W.3*

Received for publication December 5, 1963

NODES and Reid (1963) showed that the levels of certain acid-soluble nucleotides are abnormal in precancerous liver and in primary hepatomas induced by 3′-methyl-4-dimethylaminoazobenzene (3′-Me-DAB). The present paper on enzymes concerned in the metabolism of uridine nucleotides amplifies brief reports by Reid (1962*a*, *b*). The reactions now examined are shown diagramatically in Fig. 1 of the paper by Nodes and Reid (1963) and again, together with the results obtained, in the Discussion which concludes the present paper and which seeks to correlate the various findings.

### EXPERIMENTAL

A full description of the general techniques for preparing and handling the tissues, and of the historical appraisal of the tissues, has been given by Nodes and Reid (1963). Homogenates were usually prepared in 0·25 M sucrose medium and, when supernatant fractions were required, centrifuged for 90 minutes at 20,000 **g**. or, in a few experiments where a higher force was possibly advantageous, for 60 minutes at 105,000 **g**.

Abbreviations are usually restricted to standard ones as used, for example, in the *Biochemical Journal*; certain other abbreviations as used in the section "Assay of enzymic activities" are defined in that section.

### *Labelled Compounds*

The Radiochemical Centre, Amersham, supplied L-[$^{14}$C]aspartic acid (27·5 $\mu$c/mg., generally labelled), [6-$^{14}$C]orotic acid (monohydrate, 55 $\mu$c/mg.; [4-$^{14}$C]orotic acid by *Chemical Abstracts* nomenclature), and [2-$^{14}$C]uracil (i.e. uracil labelled in the carbon between the two N atoms). For the purpose of *in vitro* assays, these labelled compounds were diluted with the unlabelled compounds as supplied by British Drug Houses, Poole, Dorset.

No commercial source being available at the outset, [$^{14}$C]carbamylaspartic acid (ureidosuccinic acid) was prepared by the method of Nyc and Mitchell (1947) from unlabelled KCNO and generally labelled L-aspartic acid diluted 30-fold with unlabelled DL-aspartic acid; the product contained 0·105 $\mu$c/$\mu$mole of L-isomer. With unlabelled L-instead of DL-aspartic acid for dilution, acidification of the reaction mixture did not readily give the expected precipitate. Late in the investigation DL-[$^{14}$C]carbamylaspartic acid (labelled in the ureido group; 12·5 $\mu$c/mg.) became available from Nichem Inc. of Bethesda, Md., U.S.A.; it was diluted 5-fold with unlabelled DL-carbamylaspartic acid (Sigma Chemical Co.,

St. Louis, Miss., U.S.A.). Carbamylaspartic acid was found to be highly unstable if stored in solution, even at —20° and at neutral pH.

To obtain labelled uridine, labelled UMP synthesized enzymically from [6-$^{14}$C]orotic acid was dephosphorylated by incubation with snake venom (Russell Viper), and the reaction products were separated by chromatography of the deproteinized incubation mixture on Dowex 1 resin. In later assays of uridine kinase use was made of [$^{14}$C]uridine supplied by Schwartz BioResearch, Orangeburg, N.Y., U.S.A. ; its labelling was " general " (2·1 $\mu$c/mg.) but not uniform, being mainly in the ribose as also found by Gaito and Prusoff (1962). It was diluted with unlabelled uridine before use.

UDPglucose labelled in the glucose moiety was prepared by incubating UTP with [$^{14}$C]glucose-1-phosphate, in the presence of UDPglucose pyrophosphorylase in the form of crude " Zwischenferment " prepared from dried brewer's yeast (LePage and Mueller, 1949). The [$^{14}$C]glucose-1-phosphate was prepared by exhaustive digestion of generally labelled starch (0·4 $\mu$c/mole ; kindly provided by Dr. R. J. Bayly of the Radiochemical Centre) with phosphorylase (Sigma) in phosphate buffer. A solution of UTP (0·3 m-mole), labelled glucose-1-phosphate (0·1 m-mole), ATP (0·2 m-mole) and Zwischenferment (equivalent to 8 g. dried yeast), in 20 ml. 0·015 M K phosphate buffer (pH 7·2) containing $MgCl_2$ (0·005 M), was incubated at 30° for 30 minutes, by which time the reaction had apparently reached equilibrium. After deproteinization with $HClO_4$, and removal of $ClO_4^-$ ions as $KClO_4$, the products were chromatographically separated on Dowex 1 resin (20 cm. column). Glucose-1-phosphate (together with any glucose 6-phosphate) was eluted in advance of UMP. UDPglucose was eluted as a sharp peak, with uniform specific radioactivity through the peak ; there was negligible breakdown to glucose-1-phosphate during the final sublimation of the ammonium formate from the freeze-dried effluent.

These procedures gave [$^{14}$C]UDPglucose in a yield (based on glucose-1-phosphate) of the order of 10 per cent. It appeared that the pyrophosphorylase preparation deteriorated if stored even for a few days (as a suspension in $(NH_4)_2SO_4$ solution at −3°), and that the yield of UDPglucose could be improved if, with a view to removing the pyrophosphate formed in the reaction, inorganic pyrophosphatase (equivalent to 1 g. dried yeast) were added to the incubation medium. The pyrophosphatase was obtained from dried brewer's yeast (Heppel, 1955); the precipitate obtained by $(NH_4)_2SO_4$ was used without further purification, since it attacked ATP or UTP only slowly in comparison with inorganic pyrophosphate. Alternative methods for the preparation of [$^{14}$C]UDPglucose have been reported, but with few details (Leloir, Olavarría, Goldemberg and Carminatti, 1959 ; Robbins, Traut and Lipmann, 1959).

### *Assay of enzymic activities*

In each assay, the tissue preparation, buffer (the pH value stated being for room temperature), activators, substrate concentration, incubation time and other variables were chosen with a view to obtaining activity which was optimal in rate and was linear with respect to tissue concentration and time. Usually the literature gave some guidance, although it was sometimes found advisable to use shorter incubation times than those published ; the activities now found in normal rats were of the same order as published values. The following layout, given together with notes on some general techniques, is used for each enzyme or enzyme system :

(1) Reaction number (cf. Fig. 1 in Nodes and Reid, 1963), and International Union of Biochemistry *Code Number* if one exists. (2) *References* of particular relevance to the method adopted. (3) *Tissue addition :* nature of preparation (hom.= homogenate including nuclei, cyt.= cytoplasm, mit.= mitochondrial fraction, mic.= microsomal fraction, sup.= supernatant fraction), approximate amount as equivalent of fresh liver, and volume in which added. (4) *Other additions.* (5) *Incubation :* temperature if not 37° (the tubes being constantly shaken if risk of particles settling), time, and final treatment; $HClO_4$ (or " TCA ") denotes the treatment of the chilled incubation mixture with perchloric acid (or trichloroacetic acid), added to give a final concentration of 5 per cent w/v, followed by removal of the protein precipitate on a bench centrifuge. (6) *Final analysis,* on the protein-free supernatant or an aliquot thereof : usually by measuring ultraviolet extinction (E), or by determining inorganic orthophosphate (add 1 vol. of 5 per cent $HClO_4$-0·6 per cent ammonium molybdate, followed by reduction with ascorbic acid and measurement of $E_{720}$), or by chromatography with formic acid (FA)-ammonium formate (AmF) systems on 6·5 × 1 cm. columns of Dowex-1 resin either manually with no solvent gradient or on a fraction collector (5 ml. collections) with a discontinuous solvent gradient (cf. Hurlbert, Schmitz, Brumm and Potter, 1954) and with 125 ml. of water in the mixing flask initially. Where " plate and count " is specified, the amount of reaction product is determined by plating the material—usually a portion of column effluent—at infinite thinness and measuring the radioactivity in an end-window counter (Reid, 1964). Before chromatography on Dowex-1, $HClO_4$ supernatants were sometimes (where indicated below) heated at 100° for 20 minutes to decompose, to UMP, uridine nucleotides other than UMP. All $HClO_4$ supernatants to be chromatographed were neutralized (made alkaline to phenol red) with KOH solution and freed from the $KClO_4$ which precipitated on standing at 0°. (7) *Remarks*, especially on possible improvements in the method ; where mention is made of a " pulp ", this was liver tissue expressed through a squeezer before homogenizing (cf. De Lamirande, Daoust and Cantero, 1961).

*Aspartic transcarbamylase.*—(1) Step 1 ; 2.1.3.2. (2) Reichard (1954) ; Calva, Lowenstein and Cohen (1959) ; Smith and Baker (1959). (3) Hom. or cyt. (can be stored several months at −20°), = *c.* 70 mg., in 0·35 ml. To determine the non-enzymic condensation of the carbamylphosphate and aspartate (*c.* 10 per cent of the enzymic condensation), run a blank with the tissue addition just before the final $HClO_4$ addition. (4) 5 μmoles freshly dissolved carbamylphosphate (Sigma, crude lithium salt ; 1·53 mg.) in 0·15 ml. 0·33 M pH 8·0 tris ; 3 μmoles [$^{14}$C]aspartate (0·25μc) in 0·05 ml. water. (5) 20 min. (not linear if longer) ; finally add *c.* 1 μmole unlabelled carbamylaspartate as " carrier ", then $HClO_4$. (6) Dowex-1, manual, avoiding prolonged delay before loading (neutral solution can be kept *one* night at −20°) : apply 42 ml. of 0·05 M AmF adjusted to pH 3·2 with FA, to elute the aspartate (last 7 ml. of effluent should have negligible radioactivity), then 12 ml. 6 N FA to elute the carbamylaspartate. Plate and count. (7) Chromatography on Dowex-50 resin would be simpler (Bresnick, 1962).

*Dihydro-orotase (carbamylaspartate dehydrase) and dihydro-orotate dehydrogenase.*—(1) Step 2 ; 3.5.2.3 and 1.3.3.1. (2) Wu and Wilson (1956) ; Smith and Baker (1959) ; cf. Bresnick and Hitchings (1961) and Stevens and Stocken (1963). (3) For " free " activity, fresh hom. or cyt., = *c.* 100 mg., in 0·5 ml.

0·25 M sucrose. For " total " activity, hom. or cyt. (can be stored at −20°, even 2 years), = *c.* 60 mg., diluted to 0·5 ml. with water and either frozen and thawed altogether 8 times (activity tended to be lower if 5 or 12 times, and with only one freeze-thaw in isotonic medium was only 30 per cent above the " free " activity), or pre-incubated for 10 minutes in presence of Triton-X-100 (0·25 per cent). No blank is necessary. (4) 0·8 $\mu$moles freshly dissolved [$^{14}$C]carbamylaspartate (= 0·1 $\mu$C of L-isomer radioactivity) in 0·15 ml. 0·33 M pH 8·0 tris. (With 0·8 $\mu$moles the enzyme system was almost saturated. With pH 5·7 acetate buffer the activity was only 20 per cent of that with pH 8·0 tris.). (5) 50 min. (free activity) or 80 minutes (total activity)—not linear if longer. Finally add *c.* 1 $\mu$mole unlabelled orotate as a " carrier " and " marker ", then $HClO_4$. (6) Dowex-1, manual : apply 40 ml. 1 N FA to remove the carbamylaspartate, 5 ml. 4 N FA, and successive 6 ml. portions of 4 N FA-0·4 M AmF. Orotate (as detected by $E_{260}$ and $E_{275}$ readings) usually comes off with the 3rd or 3rd and 4th 6 ml. portions, and the preceding portions should have negligible radioactivity. Plate and count.

*Orotidylate pyrophosphorylase and orotidylate decarboxylase.*—(1) Step 3 ; 2.4.2.10 and 4.1.1.23. (2) Hurlbert and Reichard (1955) ; Herbert, Potter and Hecht (1957). (3) *Either* fresh supernatant from centrifugation of mit., i.e. mic. + sup., *or* — if only total uridine nucleotide formation being measured, the final $HClO_4$ supernatant then being heated at 100°— sup. only (can have been kept several months at −20°), = *c.* 200 mg., in 1 ml. (4) 20 $\mu$moles $KH_2PO_4$, 6·7 $\mu$moles $MgCl_2$, 1·1 $\mu$moles ATP (Sigma, K salt), 1·1 $\mu$moles ribose-5-phosphate and 2·2 $\mu$moles hexose diphosphate (each as the K salt, prepared from the Ba salt), 90 $\mu$moles nicotinamide, and 0·22 $\mu$moles [6-$^{14}$C]orotate (0·4 $\mu$C ; with 0·22 $\mu$mole the enzyme system was almost saturated — all dissolved in 1·1 ml., with addition of KOH to give pH 7·2. 0·2 $\mu$moles NAD, in 0·1 ml. (The addition in some experiments of 2 $\mu$moles glucose-1-phosphate did not markedly increase the amount of UDPglucose formed.) (5) 30°, 12 minutes (may not be linear if longer) ; finally add *c.* 0·3 $\mu$mole each of unlabelled UMP, UDP, UTP and UDPglucose as " carriers " and " markers ", then $HClO_4$. If only total uridine nucleotide formation is to be measured, heat the $HClO_4$ supernatant at 100°. (6) Dowex-1, gradient : 30 tubes with 6 N FA, UMP (detected by $E_{260}$ measurements) being usually in tubes 19–23 followed by orotate (verify that no orotate radioactivity overlaps the UMP). Then, if individual uridine nucleotides are to be measured, collect a further 30 tubes with 4 N FA-1·5 M AmF : the UDPglucose peak is typically in tube 37 (verify that no orotate radioactivity immediately precedes it), the UDP peak in tube 42, and the UTP peak in tube 50. Plate and count the effluents containing uridine nucleotides, and also—if a measure of uridine or uracil formed by catabolism of the nucleotide products is desired—the original washings and initial effluent (tubes 1 and 2), containing material not adsorbed on the column. (7) Of the total radioactivity recovered in uracil compounds, on the average about one-fifth was in each of the products (UMP, UDP, UTP, UDPglucose and uridine/uracil) ; but the relative amounts varied from one experiment to another. Liver " pulp " was only 32 per cent as active as whole liver (mean of 4 experiments), and the residue from the squeezer showed little activity.

*Uridine kinase*—(1) Step 5 ; 2.7.1.21 if identical with bacterial thymidine kinase. (2) Reichard and Sköld (1958). (3) Sup. (can have been kept several months at $-20°$), = *c*.80 mg., in 0·4 ml. (4) 5 μmoles $MgCl_2$, 3·2 μmoles ATP (K salt), 6·7 μmoles phosphoglycerate (K salt, prepared from Ba salt), and 0·5 μmole [$^{14}$C]uridine (= 0·05 μc ; position of label immaterial), in 0·15 ml. 0·1 M pH 7·4 tris. (5) 20 minutes (may not be linear if longer) ; finally add *c.* 0·2 μmole each of unlabelled UMP, UDP, UTP and UDPglucose, then $HClO_4$ ; heat supernatant at 100°. (6) Dowex-1, manual : 25 ml. 4 N FA to elute uridine, then 4 portions (10, 12, 12 and 10 ml.) of 6 N FA to elute UMP as shown by $E_{260}$ measurements—usually in the 3rd and 4th portions ; there should be no radioactivity in the preceding effluent. Freeze-dry the UMP-containing effluent, dissolve residue in 2 ml. water, plate and count.

*UTP-dephosphorylating enzyme(s)*.—(1) Step 11. (2) No literature for liver. (3) For " free " activity, fresh hom. or cyt., = *c*.2 mg., in 0·4 ml. 0·25 M sucrose. For " total " activity, hom, or cyt. (can be stored several months at $-20°$), = 2 mg. diluted to 0·4 ml. with water and frozen and thawed once ; duplicates are desirable. (4) 1·2 μmoles UTP (K salt ; enough to almost saturate the enzyme(s)) and 5 μmoles $MgCl_2$ (activity no lower if 0·5 μmole, but 25 per cent lower if 50 μmoles), in 0·1 ml. 0·3 M pH 7·4 tris. Run blanks without UTP, and with UTP but no tissue. (5) 30°, 12 minutes (" free " activity may not be linear if longer), finally $HClO_4$. (6) Determine inorganic orthophosphate. (7) Dowex-1 chromatography of reaction products from an assay of total activity with a prolonged incubation time (20 minutes) showed both UMP and UDP, the latter predominating.

*5′-UMP-dephosphorylating enzymes*.—(1) Step 6 ; one enzyme possibly 3.1.3.5 (requires $Mg^{2+}$ ions), and a second enzyme (probably identical with acid phosphatase) which is less active and does not require $Mg^{2+}$ ions. (2) De Lamirande, Allard and Cantero (1958) ; A.B.A. El-Aaser and E. Reid, unpublished experiments. (3) Hom. or cyt. (trend of results the same, whichever used, although there is some activity in nuclei), = *c*.20 mg., treated as for assay of UTP dephosphorylation ; duplicates are desirable. (4)–(6) as for above assay of UTP dephosphorylation, with UMP in place of UTP.

*Uridine phosphorylase*.—(1) Step 4 ; 2.4.2.3. (2) Reichard and Sköld (1958). (3) Sup. (can have been frozen once only, and kept at $-20°$ for several weeks), = *c*. 8 mg., in 0·4 ml. (Sup. is as active as cyt., and need not be dialyzed —cf. Reichard and Sköld, 1958). (4) 0·15 ml. 0·2 M pH 7·4 K phosphate buffer ; 5 μmoles uridine in 0·15 ml. water (activity no higher if 30 μmoles), added *after* incubation in case of blanks. (5) 60 minutes, the $HClO_4$ (2·3 ml.). (6) To 2 ml. aliquot add 2 ml. N NaOH, and determine $E_{285}$. To convert the $E_{285}$ difference (experimental minus blank ; 1 cm. light path) to μmoles uracil formed in the incubation, the factor 1·2 is used. (7) It is advisable to run both experimentals and blanks in triplicate. For linearity the $E_{285}$ difference should not exceed 0·2.

*Dihydrouracil dehydrogenase* (*uracil reductase*).—(1) Step 10 ; 1.3.1.2. (2) Fritzon (1960). The dihydrouracil formed in the assay is rapidly degraded to β-alanine, and the latter is measured. (3) Fresh sup. (freezing may destroy the activity), = *c*. 8 mg., in 0·03 ml. (the 0·25 sucrose medium may contain 0·01 M pH 8·0 tris and 0·001 EDTA) ; duplicates are desirable, but blanks are unnecessary provided that the rats had not received any isotope injection. (4) To sup. and exactly 0·025 ml. 0·15 M pH 7·4 phosphate buffer containing 0·07 μmole [6-$^{14}$C]uridine (= 0·003 μc ; " 6 " refers to the carbon atom designated " 4 " in *Chemical Abstracts*). Incubate for 15 minutes to allow uridine phosphorylase to convert uridine to uracil (not necessary if labelled uracil used in place of uridine). Then add 0·5 $\mu$mole glucose-6-phosphate (di-K or -Na), 0·3 $\mu$moles ATP, 7 $\mu$moles nicotinamide and 3 $\mu$moles NaF in 0·01 ml. water to which $MgCl_2$ (to 0·2 M) had been added just previously, and then 0·1 $\mu$mole NADP and 0·02 $\mu$mole $NADPH_2$ in 0·01 ml. water. (5) 37°, 12 minutes or, for hepatomas, 8 minutes. Finally immerse tubes in boiling water-bath for 2 minutes, and centrifuge. (6) Apply supernatant (amount need not be measured) to Whatman No. 1 chromatographic paper, and run with *n*-butanol-water (8·6 : 1·4 by vol.) until solvent front about 35 cm. from origin. Dry, and cut out (a) strip from position of original spot up to 4 cm., containing $\beta$-alanine, and (b) strip in position corresponding to uridine and uracil markers ($R_F$ values *c.* 0·25 and 0·4 respectively). Elute, plate, calculate the ratio of counts/minute in (a) to counts/minute in (a) + (b). (7) With normal or precancerous liver there is linearity up to 20 minutes of incubation, but with hepatomas there is a plateau at about 8 minutes and subsequent falling-off in apparent activity, of unknown cause (not decarboxylation of the $\beta$-alanine) ; accordingly, for some of the hepatomas the activity may have been under-estimated. The possibility that the labelled product measured was largely 5′-UMP, rather than $\beta$-alanine, was eliminated by re-running pooled samples on Dowex-1 resin and measuring the radioactivity in the effluent containing 5′-UMP. An alternative assay procedure as used for a few of the assays, depending on measurement of the loss of radioactivity from [2-$^{14}$C]uracil (Canellakis, 1956 ; Potter, Pitot, Ono and Morris, 1960), gave rather erratic results. The activity now found in normal liver was about 3 times higher than reported by other authors.

*2′(3′)-UMP-dephosphorylating enzyme(s).*—(1) Step 9. (2) A.B.A.El-Aaser and E. Reid, unpublished experiments. (3) Hom. or cyt. (can be stored 2 years at −20°), = 80 mg., in 0·4 ml. water ; duplicates are desirable. (4)–(6) as for above assay of UTP dephosphorylation, but with uridylic acid (mixed 2′- and 3′- isomers) in place of UTP and with incubation at 37°, 120 minutes.

*UDPglucose pyrophosphorylase.*—(1) Step 12 ; 2.7.7.9. (2)–(6) see Reid (1959).

*UDPglucose dehydrogenase.*—(1) Step 13 ; 1.1.1.22. (2) Strominger, Maxwell, Axelrod and Kalckar (1957). (3) Sup. (can have been kept several months at −20°), = *c.* 30 mg., in 0·2 ml. ; duplicates are desirable. (4) 3 $\mu$moles NAD in 2·7 ml. 0·11 M pH 8·7 glycine ; 0·6 $\mu$mole UDPglucose in 0·1 ml. water, or (blank) 0·1 ml. water alone. (5) and (6) Using a cuvette with 1 cm. light path, follow the rise in $E_{340}$ at room temperature (*c.* 21°) ; an increase of unity corresponds to 0·24 $\mu$mole of UDPglucose oxidized in the total volume, the linear portion being taken for the calculation.

*UDPglucuronate glucuronyltransferase.*—(1) Step 14 ; 2.4.1.17. (2) Dutton and Storey (1954) ; Dutton (1959) ; G. J. Dutton, personal communication. (3) *Fresh* hom., = *c.* 300 mg., in 1·4 ml. ; duplicates are desirable. (4) 0·3 M pH 7·4 tris containing 0·1 M $MgCl_2$, 0·2 ml. ; 0·17 $\mu$moles *o*-aminophenol (purified by sublimation) in 0·2 ml. 0·2 per cent ascorbic acid solution, this solution being stable indefinitely at −20° : for blanks, add the *o*-aminophenol solution *after* the incubation ; 0·4 $\mu$moles UDPglucuronate (prepared from liver, or purchased ; this amount was ample for saturation), in 0·2 ml. water. (5) 23°, 9 minutes (see (7) below). Finally add 2 ml. of freshly prepared mixture (1 : 1 by vol.) of 2 M phosphoric acid and 1·25 M TCA, each previously adjusted to pH 2·1 with NaOH.

(6) To 2·4 ml. of supernatant, add 0·6 ml. 0·05 per cent $NaNO_2$, leave 1 min., add 0·6 ml. 0·4 per cent ammonium sulphamate, leave 2 min., and add 0·6 ml. 0·1 per cent naphthalenediamine dihydrochloride. Keep 2 hours at room temperature in the dark, centrifuge if necessary to clarify, and determine $E_{550}$. A value of 4·5 (experimental minus blank ; 1 cm. light path) would represent 1 $\mu$mole of *o*-aminophenol glucuronide in the original incubation mixture, as shown by adding the authentic glucuronide (kindly provided by Dr. G. J. Dutton) to this mixture ; the extinction increased linearly with concentration up to $E_{550} = 1{\cdot}4$. (7) Unlike the assays performed with homogenates by Dutton and Storey (1954), the present assays showed linearity with respect to time, although even at 20° there was tailing off at 14 minutes. Liver pulp from rats fed azo dye showed similar activity to whole liver. The endogenous UDPacetylglucosamine and ATP of the fresh homogenates may, with the short incubation time employed, have been adequate to fully activate the enzyme (cf. Pogell and Leloir, 1961) ; but supplementation with these nucleotides was not tried.

*UDPglucose-glycogen transglucosylase.*—(1) Step 15 ; 2.4.1.11. (2) Leloir *et al.* (1959) ; Robbins *et al.* (1959). (3) Fresh hom. (activity low if frozen) containing 0·01 M EDTA, = *c.* 1 mg., in 0·06 ml. ; triplicates are desirable, but blanks are unnecessary. (4) 0·025 ml. of 5 per cent glycogen (British Drug Houses Ltd.) in 0·15 M pH 8·3 tris-maleate buffer containing 0·012 M EDTA ; 0·23 $\mu$moles (0·02 $\mu$c) glucose-labelled [$^{14}$C]UDPglucose in 0·015 ml. 0·033 M glucose-6-phosphate solution (K salt, prepared from Ba salt). (5) 30°, 30 minutes. Finally add 0·9 ml. 33 per cent (w/v) KOH solution, heat at 100° for 20 minutes, add 1·3 ml. ethanol, centrifuge, and wash the glycogen precipitate with a mixture of N $NH_3$ solution and ethanol (1 : 1·3 by vol.). (6) Dissolve precipitate at 37° in 1·0 ml. water, and plate. (7) Liver " pulp " was almost as active as whole liver. Addition of glucose-1-phosphate (unlabelled) did not lower the activity, thereby confirming that phosphorylase action on any glucose-1-phosphate derived from the UDPglucose was not contributing to the observed activity. Assays on sub-cellular fractions showed no activity in nuclear fractions, and a variable distribution between cytoplasmic particles (mitochondria and microsomes) and supernatant, in accord with evidence (Leloir and Goldemberg, 1960 ; Luck, 1961) that the enzyme is loosely linked to glycogen particles. In agreement with Leloir *et al.* (1959, 1960), magnesium ions were without effect or even inhibitory. The activity might have been slightly higher if glycine buffer had been used (Leloir and Goldemberg, 1960).

### RESULTS

As will be evident from the tables, there was usually no marked difference in the results between " hyperplastic nodules " and hepatomas, or between hepatomas differing in histological characteristics. It is particularly noteworthy that hepatomas with so-called " necrosis " were as high in their content of synthetic enzymes as hepatomas with little necrosis. The changes in precancerous liver were similar for different lobes of the liver (not shown in the tables).

*Synthesis of uridine-5′-monophosphate* (*UMP*).—In agreement with Calva, Lowenstein and Cohen (1959), primary hepatomas had an increased level of aspartic transcarbamylase (step 1, Table I). However, with 21–41 days' feeding of the carcinogenic azo dyes there was a slight fall in activity. With a shorter feeding period (3′-Me-DAB) the level did tend to be high, as in hepatomas, but this trend is evidently unrelated to the carcinogenicity of the dye (Table I).

TABLE I.—*Enzymes Concerned in Synthesis of Uridine 5′-Monophosphate*

In this and subsequent Tables, the mean experimental values are tabulated relative to controls taken as 100, all activities having first been calculated as μmoles/g./min. Values following the symbol ± represent standard errors. (In parentheses: number of observations and, where appropriate, the probability *P* that the difference from controls could be due to chance.) The numbering of the steps is as in Fig. 1 of the paper by Nodes and Reid (1963).

| | Carbamylphosphate → carbamylaspartate (step 1) | Carbamylaspartate → orotate (step 2) "Free" | Carbamylaspartate → orotate (step 2) "Total" | Orotate → 5′-UMP (step 3) | Uridine → 5′-UMP (step 5) |
|---|---|---|---|---|---|
| Mean value in controls μmoles/g./min. | 0·5 (=100) | 0·0022 (=100) | 0·0055 (=100) | 0·007 (=100) | 0·020 (=100) |
| *Liver from rats fed 2-Me-DAB (virtually non-carcinogenic)* | | | | | |
| 12–19 days . . . | 144 (2) | 79 (1) | | 89 (1) | 88 (4) } 87 ±8 |
| 35–41 days . . . | 81 (1) | 120 (1) | | 72 (1) | 80 (1) } |
| *Liver from rats fed 4′-Me-DAB (virtually non-carcinogenic)* | | | | | |
| 12–19 days . . . | 230 (2) | 114 (1) | | 95 (1) | 102 ±32 (4) |
| 24–41 days . . . | 107 (2) | 80 (2) | | 145 (3) } 165 ±23 (P< 0·1) | 90 (2) |
| 3 months, then 3 months off dye . . . | | | | 224 (1) } | 100 (1) |
| *Liver from rats fed 3′-Me-DAB (highly carcinogenic)* | | | | | |
| 3 days . . . | | | | 113 (2) } 131 ±13 (P< 0·05) | |
| 5–20 days . . . | 170 ±51 (4) | 150 ±19 (7; P<0·05) | 140 (3) } 128 ±22 | 118 (9) } | 170 (9) } 153 ±15 (P< 0·005) |
| 21–45 days . . . | 73 ±8 (8; P<0·025) | 104 ±14 (4) | 123 (8) } | 154 (7) } | 138 (7) } |
| 80 days . . . | | | | 73 (1) } | |
| *Liver from rats fed 4′-F-DAB (highly carcinogenic)* | | | | | |
| 12–20 days . . . | 111 ±15 (5) | 69 (3) | | 141 (3) } 134 ±8 (P< 0·025) | 126 ±5 (7; P<0·005) |
| 21–35 days . . . | 55 (2) } 70 ±11 (P< 0·1) | | 93 <30 (4) | 125 (2) } | 65 ±13 (4; P<0·1) |
| 36–51 days . . . | 87 (2) } | 120 ±10 (5; P<0·1) | | | 229 (3) |
| *"Normal" liver adjoining nodules induced by 3′-Me-DAB* | | | | 183 (3) | 83 (3) |
| *Nodules induced by 3′-Me-DAB* | | | | | |
| Hepatoma nodules . | 187 ±22 (6; P<0·025) | 163 ±26 (11; P<0·05) | 191 ±35 (12; P<0·025) | 33 ±8 (5; P<0·001) | 274 ±38 (6; P<0·005) |
| *Hepatoma sub-categories:* | | | | | |
| Metastases . . | 204 (1) | 142 (1) | 200 (1) | | 475 (1) |
| Necrosis limited . | 150 (2) | 134 (5) | 152 (6) | 30 (4) | 244 (4) |
| Necrosis very extensive | 179 (2) | 180 (4) | 208 (4) | 32 (1) | 240 (1) |
| Adenocarcinoma . | 133 (2) | 227 (5) | 200 (2) | 33 (3) | 280 (1) |
| Trabecular carcinoma | 192 (2) | 160 (2) | 161 (2) | 44 (1) | 280 (3) |
| Mainly small-celled . | | | 141 (2) | | 140 (1) |
| Mainly large-celled . | 180 (1) | 136 (3) | 190 (4) | 36 (4) | 253 (2) |
| Leucocytes abundant | 120 (1) | 103 (2) | 182 (1) | | 140 (1) |
| Hyperplastic nodules . | 203 (2) | | 270 (2) | 21 (2) | 384 (2) |
| *Hepatoma and hyperplastic nodule sub-category:* | | | | | |
| Extensive fibrosis . | 197 (4) | 160 (6) | 184 (9) | 35 (4) | 285 (3) |

The next step in the *de novo* pathway for synthesis of UMP showed, both for " free " and for " total " activity, high values in hepatomas and, less strikingly, in precancerous liver (step 2, Table I). The further step whereby orotate is converted into UMP showed impaired activity in hepatomas, but somewhat enhanced activity in precancerous liver (step 3, Table I). A similar but possibly later enhancement tended to occur with the non-carcinogenic dye 4′-Me-DAB. These results for UMP formation take account of the partial transformation of the UMP into products such as UTP ; there were no clear-cut effects of carcinogenesis on the pattern of these secondary conversions (not shown in the table).

The enzyme uridine kinase, lying on the " salvage " pathway of UMP synthesis, showed a striking rise in hepatomas, and a moderate rise at an early stage of feeding with the carcinogenic azo dyes (step 5, Table I). The rise was possibly bi-phasic in the case of 4′-F-DAB.

*Catabolism of uridine nucleotides.*—The enzyme(s) concerned in dephosphorylation of UTP show, at least with respect to " free " activity, enhanced activity in hepatomas and in liver from rats fed carcinogenic azo dyes for more than three weeks (step 11, Table II).

The enzymes which effect the dephosphorylation of 5′-UMP in the presence of $Mg^{2+}$ ions showed no significant change in " total " activity other than a small and transient fall in rats fed 3′-Me-DAB (step 6, Table II). The activity as measured is due mainly to an enzyme located in nuclei and microsomes and in small part to an enzyme—probably acid phosphatase—which is located in lysosomes, is " latent " in fresh homogenates, and does not require $Mg^{2+}$ ions (A. B. A. El-Aaser and E. Reid, unpublished experiments). In experiments not tabulated the two enzymes were assayed individually, by using fresh homogenates so that only " free " activity was measured and by omitting $Mg^{2+}$ ions respectively. The results summarized by Reid (1962*a*, *b*) for precancerous liver were in fact mainly preliminary assays performed without $Mg^{2+}$ ions, the importance of which was not then appreciated. The further work now performed has established that 5′-UMP dephosphorylation, whatever the assay procedure, is not consistently enhanced in precancerous liver or hepatomas.

With 2′-(3′-)UMP as substrate, variable but usually enhanced dephosphorylation was found in hepatomas (step 9, Table II) ; however, for some of the hepatomas studied in these experiments, and for most of those studied more recently (A. B. A. El-Aaser and E. Reid, unpublished experiments), the activity was normal or even somewhat low. The further finding that uridine phosphorylase was abnormally high in hepatomas (step 4, Table II) is not of great interest when viewed in the light of the apparent falling-off of the normal activity with age (see footnote to Table II).

The enzyme dihydrouracil dehydrogenase (uracil reductase) showed somewhat depressed activity in rats fed azo dyes—even non-carcinogenic azo dyes—and variable but, on the average, normal activity in hepatomas (step 10, Table II). Unexplained variability in this activity has likewise been encountered, with Hepatoma 5123 transplants, by Ono, Blair, Potter and Morris (1963).

*Metabolism of conjugated uridine nucleotides.*—As is shown in Table III (step 12), the activity of UDPglucose pyrophosphorylase, whereby UTP is converted into UDPglucose, was somewhat depressed in hepatomas, and also in liver from rats fed 3′-Me-DAB or non-carcinogenic azo dyes but not 4′-F-DAB. UDPglucose dehydrogenase (step 13) was likewise moderately depressed in precancerous liver, apparently by the carcinogenic dyes specifically, but was usually unaltered in hepatomas.

The transglucuronylase which converts *o*-aminophenol into its glucuronide showed high values in liver from rats fed azo-dyes, carcinogenic or non-carcino-

TABLE II.—*Enzymes Concerned in Catabolism of Uridine Nucleotides*

| | UTP dephosphorylation (step 11) "Free" | UTP dephosphorylation (step 11) "Total" | 5′-UMP dephosphorylation (step 6) | 2′-(3′-)UMP dephosphorylation (step 9) | Uridine →uracil (step 4) | Uracil →dihydrouracil (step 10) |
|---|---|---|---|---|---|---|
| Mean value in controls, $\mu$moles/g./min. | 6 (=100) | 15 (=100) | 5 (=100) | 0·35 (=100) | 0·4* (=100) | 0·14 (=100) |
| *Liver from rats fed 2-Me-DAB (virtually non-carcinogenic)* | | | | | | |
| 12–19 days . . | | | 85 (2) } 85 ±6 (P< 0·1) | | | 30 (1) |
| 35–51 days . . | 136 (3) | | 85 (3) | | | 67 (1) |
| *Liver from rats fed 4′-Me-DAB (virtually non-carcinogenic)* | | | | | | |
| 12–19 days . . | | | 134 (2) | | | 38 (1) |
| 24–51 days . . | 92 (3) | | 92 (3) | | 45 (1) | 83 (2) |
| *Liver from rats fed 3′-Me-DAB (highly carcinogenic)* | | | | | | |
| 5–20 days . . . | 98 (2) | 90 ±14 (5) | 78 ±6 (5; P<0·025) | 100 (3) | 92 (8) } 92 ±7 | 56 (2) } 44 ±11 (P< 0·025) |
| 21–45 days . . | 176 ±26 (5; P< 0·05) | 100 (1) | 101 (3) | 120(2) | 91 (6) | 57 (3) |
| *Liver from rats fed 4′-F-DAB (highly carcinogenic)* | | | | | | |
| 12–19 days . . | | | 97 (2) | | | 78 (1) } 71 ±3 (P< 0·005 |
| 35–51 days . . | 160 ±20 (5; P< 0·05) | | 92 (3) | | | 69 (3) |
| *" Normal " liver adjoining nodules induced by 3′-Me-DAB* | | | | | | |
| | | | | | 243 ±35 (4; P< 0·05) | |
| *Nodules induced by 3′-Me-DAB* | | | | | | |
| Hepatoma nodules . | 145 ±9 (6; P< 0·005) | 126 ±12 (10; P< 0·1) | 128 ±14 (16; P< 0·1) | 168 ±30 (11; P< 0·05) | 380 ±59 (7; P< 0·025) | 98 ±16 (10) |
| *Hepatoma sub-categories :* | | | | | | |
| Metastases . . | 162 (1) | 77 (1) | 58 (1) | 440 (1) | | |
| Necrosis limited . | 150 (3) | 119 (4) | 131 (8) | 138 (6) | 380 (7) | 96 (4) |
| Necrosis very extensive | 145 (2) | 143 (3) | 122 (5) | 160 (3) | | 83 (3) |
| Adenocarcinoma . | 174 (1) | 113 (2) | 198 (3) | 132 (3) | 350 (4) | 106 (2) |
| Trabecular carcinoma | 126 (2) | 130 (6) | 111 (9) | 204 (6) | 323 (2) | 141 (1) |
| Mainly small-celled . | | 182 (1) | 116 (1) | 138 (1) | | |
| Mainly large-celled . | 145 (2) | 127 (2) | 119 (4) | 130 (3) | 402 (5) | 80 (1) |
| Leucocytes abundant | 174 (1) | 141 (2) | 160 (3) | 155 (3) | | 185 (1) |
| Hyperplastic nodules . | | 210 (3) | 101 (3) | 138 (3) | 600 (2) | 114 (3)† |
| *Hepatoma and hyperplastic nodule sub-category :* | | | | | | |
| Extensive fibrosis . | 147 (5) | 120 (6) | 122 (11) | 190 (7) | 205 (2) | 91 (4) |

* Excluding old rats (controls for rats with nodules), for which the mean value was 0·15—an age difference converse to that reported by Stevens & Stocken (1963) : the apparently high values found in the nodules were in fact no higher than the values for young controls.

† Excluding 3 cholangiomas, 2 of which showed markedly increased values.

genic (step 14, Table III). Hepatomas showed an average activity close to normal.

UDPglucose is the source not only of UDPglucuronate and thence of glucuronides, but also of glycogen. The transglucosylase concerned in glycogen formation showed low activity in hepatomas, and perhaps a slight fluctuation—a rise followed by a fall—in precancerous liver (step 15, Table III).

### DISCUSSION

The histological findings (Nodes and Reid, 1963) in conjunction with the biochemical findings suggest that parenchymal cells are the site of the changes observed in the present enzymic assays on precancerous liver. One enzyme in

TABLE III.—*Enzymes Concerned in Metabolism of Conjugated Uridine Nucleotides*

| | UDPglucose pyrophosphorylase (step 12) | UDPglucose dehydro-genase (step 13) | UDPglucuronate transglucuronylase (step 14) | UDPglucose-glycogen trans-glucosylase (step 15) |
|---|---|---|---|---|
| Mean value in controls, μmoles/g./min. | 0·4 (=100) | 0·08 (=100) | 0·005 (=100) | 0·9 (=100) |
| *Liver from rats fed 2-Me-DAB (virtually non-carcinogenic)* | | | | |
| 19 days | 69 (2) } 73±4 ($P<0·01$) | | 136 (2) } 145±15 ($P<0·05$) | |
| 35–41 days | 77 (2) | | 149 (4) | |
| *Liver from rats fed 4′-Me-DAB (virtually non-carcinogenic)* | | | | |
| 19 days | 65 (2) | 92 (2) | 107 (2) } 181 ±61 | |
| 24–41 days | 106 (1) | 176 (3) | 210 (5) | |
| *Liver from rats fed 3′-Me-DAB (highly carcinogenic)* | | | | |
| 3 days | 175 (1) | 127 (2) } 118 ±11 | 17 (2) | |
| 5–20 days | 74 (4) } 77 ±9 ($P<0·05$) | 115 (7) | 127 ±38 (8) | 123 ±11 (6; $P<0·1$) |
| 21–35 days | 79 (6) | 68 ±10 (5; $P<0·05$) | 212 (7) } 214 ±46 ($P<0·05$) | 76 ±10 (6; $P<0·1$) |
| 80 days | | | 233 (1) | |
| *Liver from rats fed 4′-F-DAB (highly carcinogenic)* | | | | |
| 13–20 days | 94 ±16 (5) | 67 ±11 (5; $P<0·05$) | 100 (2) | |
| 27–41 days | 146 (3) | 117 (3) | 149 ±28 (4) | |
| *"Normal" liver adjoining nodules induced by 3′-Me-DAB* | | | | |
| | 125 (3) | 152 ±26 (4) | 72 (3) | 102 (3) |
| *Nodules induced by 3′-Me-DAB* | | | | |
| Hepatoma nodules | 59 ±14 (9; $P<0·025$) | 91 ±21 (8) | 86 ±24 (7) | 36 ±13 (5; $P<0·01$) |
| *Hepatoma sub-categories :* | | | | |
| Metastases | 7 (1) | | | |
| Necrosis limited | 74 (6) | 92 (7) | 80 (5) | 36 (5) |
| Necrosis very extensive | 41 (2) | 90 (1) | 101 (2) | |
| Adenocarcinoma | 63 (3) | 115 (2) | 86 (2) | 48 (3) |
| Trabecular carcinoma | 51 (5) | 129 (3) | 78 (4) | 13 (1) |
| Mainly small-celled | | 162 (2) | 52 (2) | |
| Mainly large-celled | 69 (4) | 66 (4) | 88 (1) | 42 (4) |
| Hyperplastlc nodules | 28 (2) | 82 (3) | 33 (1) | 35 (1) |
| *Hepatoma and hyperplastic nodule sub-category :* | | | | |
| Extensive fibrosis | 52 (3) | 100 (6) | 84 (5) | 30 (2) |

the *de novo* pathway of 5′-UMP synthesis—dihydroorotate dehydrogenase—is in fact known to be present in parenchymal cells (Cohen, 1962). In some instances there appeared to be up-and-down changes, for which Reid (1962*a*) has collated other examples (e.g. Trams, Inscoe and Resnik, 1961) and suggested an explanation—that cells other than a few destined to become cancerous may " overcompensate " for initial biochemical effects of the carcinogen treatment. Allowing for such fluctuations, and for the variability sometimes encountered among primary hepatomas but seldom explicable in terms of histological differences, the findings suggest the following conclusions.

*Relevance of the enzymic findings to azo-dye carcinogenesis.*—The findings for the metabolism of conjugated uridine nucleotides will first be considered. The rise in UDPglucuronate transglucuronylase at about 3 weeks—as also found with DAB feeding (Trams *et al.*, 1961)—is evidently a non-specific response to the feeding of azo dyes, metabolites of which are excreted as glucuronides ; there is other evidence (Takemori and Glowacki, 1962) that this enzyme is rate-limiting in glucuronide synthesis. The fall in UDPglucose dehydrogenase seen during dye feeding appeared, unlike the fall in UDPglucose pyrophosphorylase, to be specifically correlated with carcinogenesis ; but in rats fed DAB (Trams *et al.*, 1961) and in the present hepatomas the dehydrogenase activity was almost normal. The fall in UDPglucose-glycogen transglucosylase in hepatomas may in part explain their well-known lack of glycogen (Reid, 1962*a*) ; but if impaired glycogen storage is a very early event in hepatocarcinogenesis (Porter and Bruni, 1959 ; Muramatsu, 1961), then the cause cannot be loss of this enzyme since it is undiminished in early-precancerous liver. These conclusions still hold if glycogen synthesis is limited not by the enzyme but by the supply of UDPglucose, data for which are discussed below.

The observations on steps in the synthesis of UMP are summarized in Fig. 1. The activities of the different steps in the *de novo* pathway (from carbamylaspartate) evidently do not change in parallel, although there is a general trend towards an increase. The three enzyme systems concerned appear to show a serial change, the first tending to be especially high with 5–20 days of feeding with 3′-Me-DAB and the third tending to be especially high with 21–41 days of feeding as if there were stepwise " induction " of the different enzymes. However, the results with 4′-F-DAB (Table I) were less clearcut. Moreover, from hormonal and other data Reid (1962*b*) argued that the overall activity of the *de novo* pathway is governed by the " free " activity of the intermediate enzyme system which converts carbamylaspartate into orotate. By this argument, the increase in this activity found in rats fed 3′-Me-DAB and in the hepatomas connotes faster operation of the whole pathway.

This conclusion may still hold if emphasis is put rather on the results for carbamylaspartate formation, a process for which a supply of carbamylphosphate is required. By virtue of the marked fall in the capacity for formation of ornithine (and ultimately of urea) from carbamylphosphate in azo-dye carcinogenesis (McLean, Reid and Gurney, 1964 ; cf. Burke and Miller, 1959), channelling of carbamylphosphate into the carbamylaspartate branch would be favoured, provided that the fall in aspartate in azo-dye carcinogenesis (Muramatsu, 1961) is not so great as to render aspartate rate-limiting (cf. Smith and Baker, 1959).

There may, then, be acceleration of the *de novo* pathway, and also of the salvage pathway as judged by the uridine kinase assays (Fig. 1). At 5 days of dye feeding uridine kinase already showed a rise in activity. Evidently the rise is not secondary to a " deletion " in the *de novo* pathway. The histological results rule out the possibility—suggested by the high uridine-kinase activity of rat bone marrow (Sköld, 1960)—that leucocyte infiltration might account for the high values in precancerous liver or hepatomas.

With regard to the catabolism of uridine nucleotides, increased " free " activity of the enzyme(s) which dephosphorylate UTP is found both in hepatomas and in liver from rats fed carcinogenic azo dyes for 3–6 weeks. The other catabolic reactions studied showed no early changes specifically linked with carcinogenic azo dyes, and no consistent decreases in the hepatomas.

FIG. 1.—Activities (per g. of tissue) of enzymes concerned with synthesis of 5′-UMP, in precancerous liver (3′-Me-DAB) and in hepatoma nodules induced by 3′-Me-DAB. Figures in parentheses represent the mean normal activity, in $\mu$moles/g./min.

*Re-examination of the enzymic findings in relation to changes in acid-soluble nucleotide and RNA levels.*—Fig. 2 shows, together with the present findings, related findings such as the changes found by Nodes and Reid (1963) in the levels of acid-soluble nucleotides and in the activities of the enzymes which break RNA down to 2′- or 3′-mononucleotides. Of the acid-soluble nucleotides, only the uracil derivatives will now be considered, since Nodes and Reid (1963) have already discussed the findings for purine nucleotides and for " pyridine " nucleotides such as NAD.

The results for UDPglucose illustrate the difficulty of deciding whether a change in the amount of a tissue constituent is due to altered synthesis or altered utilization, and whether the rate-limiting factor for a particular reaction is the enzyme level or the supply of a substrate. The level of UDPglucose rises in precancerous liver and falls in hepatomas (Fig. 2). In hepatomas the fall may be due in part to the diminished activity of the enzyme which effects the synthesis of UDPglucose from UTP and glucose-1-phosphate (the level of the latter being undiminished in azo-dye hepatomas, Lepage, 1948) ; but the cause may lie mainly in accelerated formation of mucopolysaccharide as discussed by Nodes and Reid (1963), by reactions involving UDPglucuronate derived from UDPglucose. In the case of precancerous liver, the rises in UDPglucose and in UDPglucuronate can be attributed to faster production, reflecting increased availability of 5′-UMP and meeting an increased need in connection not with building fibrous tissue as in hepatomas, but perhaps with synthesis of ascorbic acid (cf. Daff, Hoch-Ligeti,

FIG. 2.—Levels of RNA and of acid-soluble nucleotides, and activites of enzymes concerned in RNA and nucleotide metabolism :—summary of findings in this laboratory for precancerous liver (3′-Me-DAB, fed for 2–5 weeks) and for primary hepatoma nodules.

Changes (per g. tissue) in the level of RNA in a cell fraction, or of an acid-soluble nucleotide measured in whole liver, are shown *above* the entry for the constituent ; changes (per g. tissue) in the activity of an enzyme *in vitro* are shown *below* the arrow for the reaction. The ribonucleases and phosphodiesterases concerned in RNA breakdown were assayed in individual sub-cellular fractions (Nodes and Reid, 1963). For ATP-ase (Reid and O'Neal, 1956) and for other enzymes which are partly " latent ", the change shown is for the " total " activity. The findings for adenosine catabolism, for nuclear RNA, and for RNA in other fractions are those of Reid and Lewin (1957), of M. K. Turner (unpublished experiments) and of Reid (1958) respectively.

Kennaway and Tipler, 1948, and Conney, Bray, Evans and Burns, 1961) and particularly of glucuronides.*

The general rise in uridine nucleotide levels in precancerous liver is attributable to faster UMP synthesis by the *de novo* and salvage pathways (Fig. 1 and 2), from the enzymic evidence discussed above. It is unlikely that the depletion in ATP level (Fig. 2) renders ATP a limiting factor in UMP synthesis. The supply of uridine for the salvage pathway may actually be enhanced, since RNA breakdown appears to be accelerated and uracil catabolism depressed (Fig. 2). The conclusion that UMP synthesis is accelerated is supported by the finding that, in rats fed 3′-Me-DAB, UMP showed a greater rise in level than other uridine

* Note added in proof :—

At the suggestion of Dr. J. E. Scott, nodules rich in fibrous tissue have now been treated with different stains (by Mr. E. Woollard), and collagen was found to predominate over mucopolysaccharide.

nucleotides (Nodes and Reid, 1963). This preferential rise could, however, be due in part to slower catabolism of UMP, there being a fall in 5′-nucleotidase activity, and in part to impaired conversion of UMP to UDP and thence to UTP because of the fall in ATP level. One factor which would set a limit to the rise is feed-back inhibition, by UMP itself (Blair and Potter, 1961 ; Creasey and Handschumacher, 1961), of orotidylate decarboxylase, the last enzyme in the *de novo* pathway.

In the hepatomas (Fig. 2), despite the enzymic evidence for faster synthesis of UMP there was no consistent increases in the level of UMP or of UTP, although there was an increase in UDP level due probably to faster dephosphorylation of UTP. If in the hepatomas there is indeed faster synthesis of uridine nucleotides, this may be outweighed by the postulated faster consumption of UDPacetyl-glucosamine and UDPglucuronate, and by faster incorporation of UTP into RNA (Reid, 1958, and unpublished experiments).

That there may be faster breakdown of RNA to nucleotides, both in precancerous liver and in the hepatomas, is suggested by the observation of Nodes and Reid (1963) that the acid-ribonuclease activity of the supernatant fraction (as distinct from the activity bound in lysosomes) is increased, even before microsomal RNA decreases (Reid, 1964). Breakdown of the nucleotide products to give inosine or uracil (Fig. 2) is evidently undiminished, if not accelerated, even in hepatomas. The classical view that catabolic reactions are suppressed in tumours may nevertheless have some validity for *late* steps in nucleotide catabolism, since low activity has been observed for xanthine oxidase and, with some but not all of the hepatomas studied, for uracil reductase. The fall in $NADPH_2$ in the hepatomas may, however, restrict uracil reduction *in vivo* (Fritzon, 1960).

*Are any of the findings generally applicable to hepatocarcinogenesis?*—Dihydrouracil dehydrogenase, which is non-specifically depressed by azo-dye feeding, is likewise depressed by 2-acetylaminofluorene (P. Fritzon, personal communication). This carcinogen also resembles the azo-dyes now studied in causing a transient rise in glucuronyl transferase and fall in UDPglucose dehydrogenase (Trams *et al.*, 1961). Otherwise there appears to be no information concerning early effects of hepatocarcinogens other than azo dyes on the enzymic activities now studied. Information concerning these activities in primary hepatomas is also lacking, except for the report of Calva *et al.* (1959) who found increased aspartate transcarbamylase in azo-dye hepatomas as in the present study. Accordingly, for the purpose of deciding whether the changes now found in primary hepatomas are irreducible properties of hepatomas, comparison must be made with transplanted hepatomas. The Novikoff hepatoma shows low activity for UDPglucose pyrophosphorylase, for the glycogen-synthesizing enzyme and—in contrast with the hepatomas now studied—for UDPglucose dehydrogenase (Nigam, MacDonald and Cantero, 1962). However, comparison with the Novikoff hepatoma (for which Novikoff (1960) has summarized the literature) is unprofitable, since there is now ample evidence (e.g. Potter *et al.*, 1960 ; Ono *et al.*, 1963) that this hepatoma is highly uncharacteristic. Comparison can, however, advantageously be made with " minimum deviation " heptomas such as the Morris 5123.

Morris 5123 hepatomas, unlike those now studied, show no elevation of aspartate transcarbamylase activity (Ono *et al.*, 1963), a normal capacity for conversion of carbamylaspartate into orotate (Reid and Morris, 1963), and only a moderate impairment of the capacity for converting orotate into UMP (Ono *et al.*, 1963 ; Reid and Morris, 1963). Moreover, uridine kinase activity in Morris 5123 hepatomas is normal (Reid and Morris, 1963). There is, then, no evidence for accelerated formation of UMP in the Morris 5123 hepatoma ; the apparent acceleration in the azo-dye hepatomas now studied may, in accordance with a suggestion by Sköld (1960), be a reflection of the great rapidity of their growth. It is striking that abnormally high activities have been found for uridine kinase in human tumours (Kára, Šorm and Winkler, 1963) and for enzymes of the *de novo* pathway for UMP synthesis in leukaemic leucocytes (Smith, Baker and Sullivan, 1960). Moreover, there is evidence that the latter pathway is affected —whether blocked or stimulated is not clear—in urethane carcinogenesis (Rogers, 1957). Although faster UMP synthesis is not an absolute requisite for neoplastic growth, it may be a *pre*-requisite, since it was in evidence not only in the primary hepatomas but also in " hyperplastic nodules " and in precancerous liver.

One of the catabolic steps now found to be altered—the " free " capacity for dephosphorylation of UTP—may also be high in the Morris 5123 hepatoma ; but UDPglucose pyrophosphorylase activity was normal in the 5123 hepatoma (Reid and Morris, 1963). In two respects, however, the primary hepatomas may have been more " mimimum-deviation " in type than 5123 hepatomas : the latter tend to be low in UDPglucose dehydrogenase (Reid and Morris, 1963) and especially in glucuronide-synthesizing activity (Novikoff, 1960), whereas these enzymes were normal in many of the hepatomas now studied.

In general the present enzymic comparisons of primary hepatomas with normal liver have shown relatively few differences (" deviations "), but the hepatomas were evidently not of the " minimum-deviation " type in respect of all these differences. One important conclusion is that, contrary to a belief based, for example, on uracil catabolism in a mouse hepatoma (Canellakis, 1957), catabolic reactions may be undiminished in tumours.

The results presented and discussed in the preceding papers (Nodes and Reid, 1963 ; Reid, 1964) do suggest some provisional generalizations about hepatoma cells, irrespective of whether the cells appear " necrotic ". In whole tissue there are markedly lowered levels of UDPglucuronate, $NADPH_2$ (and usually NADP), and certain purine nucleotides other than ATP. The acid-soluble purine nucleotides of isolated mitochondria are depleted, as are mitochondrial protein and microsomal protein and RNA. The turnover of RNA is faster, there being acceleration both of its synthesis as judged by orotate incorporation *in vivo* and of its catabolism as judged by the acid-ribonuclease activity of the supernatant fraction. Most of the changes found in primary hepatomas are already evident at some stage early in the feeding period.

### SUMMARY

Enzymes concerned in the metabolism of uridine nucleotides have been assayed in primary hepatomas, and in liver from rats fed carcinogenic or non-carcinogenic azo dyes. The histological character of the hepatomas, e.g. the degree of necrosis, had no clear influence on the results.

Precancerous liver showed, in the *de novo* pathway for synthesis of 5′-UMP, an eventual slight decrease in " step 1 " (carbamylaspartate formation), and moderate increases in " step 2 " (orotate formation) and " step 3 " (UMP formation) ; the overall capacity for UMP synthesis is considered to be slightly increased. In the hepatomas steps 1 and 2 showed increased activity, and although step 3 showed decreased activity it is considered that the capacity for *de novo* synthesis of UMP was increased. As judged by the assays for uridine kinase, the salvage pathway for synthesis of UMP was likewise increased in azo-dye hepatomas, and in rats fed carcinogenic azo dyes.

Assays on precancerous liver for enzymes concerned in nucleotide catabolism showed an eventual rise in the " free " capacity for UTP dephosphorylation, and a decrease—apparently not specifically related to carcinogenesis—in dihydrouracil dehydrogenase. In the hepatomas the former activity was high and the latter was usually close to normal. None of the catabolic enzymes measured showed consistently low activity in the hepatomas.

UDPglucose pyrophosphorylase was somewhat low in the hepatomas, but in precancerous liver there was no change specifically related to carcinogenesis. UDPglucose dehydrogenase showed little change in hepatomas but a specific depression in precancerous liver. Glucuronide-synthesizing activity showed a non-specific rise in precancerous liver. Glycogen-synthesizing activity was low in the hepatomas.

The results are discussed in relation to their bearing on data for nucleotide levels (Nodes and Reid, 1963), and to their significance for hepatocarcinogenesis.

Thanks are expressed to Miss P. M. Law and Mr. A. Coldman for technical help, to Dr. G. J. Dutton for advice on assay of glucuronide synthesis, and Professor A. Haddow, F.R.S., for his interest in the work described here and in the preceding papers. The work was supported by grants to the Chester Beatty Research Institute (Institute of Cancer Research : Royal Cancer Hospital) from the Medical Research Council, the British Empire Cancer Campaign, the Anna Fuller Fund, and the National Cancer Institute of the National Institutes of Health, U.S. Public Health Service.

### REFERENCES

Blair, D. G. R. and Potter, V. R.—(1961) *J. biol. Chem.*, **236**, 2503.
Bresnick, E.—(1962) *Biochim. biophys. Acta*, **61**, 598.
*Idem* and Hitchings, G. H.—(1961) *Ibid.*, **21**, 105.
Burke, W. T. and Miller, L. L.—(1959) *Ibid.*, **19**, 622.
Calva, E., Lowenstein, J. M. and Cohen, P. P.—(1959) *Ibid.*, **19**, 101.
Canellakis, E. S.—(1956) *J. biol. Chem.*, **221**, 315.—(1957) *Ibid.*, **227**, 701.
Cohen, R. B.—(1962) *Lab. Invest.*, **11**, 531.
Conney, A. H., Bray, G. A., Evans, C. and Burns, J. J.—(1961) *Ann. N.Y. Acad. Sci.*, **92**, 115.
Creasey, W. A. and Handschumacher, R. E.—(1961) *J. biol. Chem.*, **236**, 2058.
Daff, M., Hoch-Ligeti, C., Kennaway, E. L. and Tipler, M. M.—(1948) *Cancer Res.*, **8**, 376.
De Lamirande, G., Allard, C. and Cantero, A.—(1958) *Ibid.*, **18**, 952.
*Idem*, Daoust, R. and Cantero, A.—(1961) *Canad. Cancer Conf.*, **4**, 43.
Dutton, G. J.—(1959) *Biochem. J.*, **71**, 141.
*Idem* and Storey, I. D. E.—(1954) *Ibid.*, **57**, 275.
Fritzon, P.—(1960) *J. biol. Chem.*, **235**, 719.
Gaito, R. A. and Prusoff, W. H.—(1962) *Biochem. Pharmacol.*, **11**, 323.
Heppel, L.—(1955) ' Methods in Enzymology ', Vol. II, 1st edition. Edited by S. P. Colowick and N. O. Kaplan. New York (Academic Press) p. 570.

HERBERT, E., POTTER, V. R. AND HECHT, L. I.—(1957) *J. biol. Chem.*, **225**, 659.
HURLBERT, R. B. AND REICHARD, P.—(1955) *Acta chem. scand.*, **9**, 251.
*Idem.*, SCHMITZ, H., BRUMM, A. F. AND POTTER, V. R.—(1954) *J. biol. Chem.*, **209**, 23.
KÁRA, J., ŠORM, F. AND WINKLER, A.—(1963) *Neoplasma*, **10**, 3.
LELOIR, L. F. AND GOLDEMBERG, S. H.—(1960) *J. biol. Chem.*, **235**, 919.
LELOIR, L. F., OLAVARRÍA, J. M., GOLDEMBERG, S. H. AND CARMINATTI, H.—(1959) *Arch. Biochem. Biophys.*, **81**, 508.
LEPAGE, G. A.—(1948) *Cancer Res.*, **8**, 193.
*Idem* AND MUELLER, G. C.—(1949) *J. biol. Chem.*, **180**, 975.
LUCK, D. J.—(1961) *J. biophys. biochem. Cytol.*, **10**, 195.
MCLEAN, P., REID, E. AND GURNEY, M.—(1964) *Biochem. J.* (in press).
MURAMATSU, M.—(1961) *Gann*, **52**, 135.
NIGAM, V. N., MACDONALD, H. L. AND CANTERO, A.—(1962) *Cancer Res.*, **22**, 131.
NODES, J. T. AND REID, E.—(1963) *Brit. J. Cancer*, **17**, 745.
NOVIKOFF, A. B.—(1960) ' Cell Physiology of Neoplasia '. University of Texas, M.D. Anderson Hospital and Tumor Institute. Austin (University of Texas Press), p. 219.
NYC, J. F. AND MITCHELL, H. K.—(1947) *J. Amer. chem. Soc.*, **69**, 1382.
ONO, T., BLAIR, D. G. R., POTTER, V. R. AND MORRIS, H. P.—(1963) *Cancer Res.*, **23**, 240.
POGELL, B. M. AND LELOIR, L. F.—(1961) *J. biol. Chem.*, **236**, 293.
PORTER, K. R. AND BRUNI, C.—(1959) *Cancer Res.*, **19**, 997.
POTTER, V. R., PITOT, H. C., ONO, T. AND MORRIS, H. P.—(1960) *Ibid.*, **20**, 1255.
REICHARD, P.—(1954) *Acta chem. scand.*, **8**, 795.
*Idem* AND SKÖLD, O.—(1958) *Biochim. biophys. Acta*, **28**, 376.
REID, E.—(1958) *Brit. J. Cancer*, **12**, 428.—(1959) *Biochim. biophys. Acta*, **32**, 251.—(1962*a*) *Cancer Res.*, **22**, 398.—(1962*b*) *Nature, Lond.*, **194**, 1153.—(1964) *Brit. J. Cancer*, **18**, 172.
*Idem* AND LEWIN, I.—(1957) *Ibid.*, **11**, 494.
*Idem* AND MORRIS, H. P.—(1963) *Biochim. biophys. Acta*, **68**, 647.
*Idem* AND O'NEAL, M. A.—(1956) *Brit. J. Cancer*, **10**, 287.
ROBBINS, P. W., TRAUT, R. R. AND LIPMANN, F.—(1959) *Proc. nat. Acad. Sci., Wash.*, **45**, 6.
ROGERS, S.—(1957) *J. exp. Med.*, **105**, 279.
SKÖLD, O.—(1960) *Biochim. biophys. Acta*, **44**, 1.
SMITH, L. H. AND BAKER, F. A.—(1959) *J. clin. Invest.*, **38**, 798.
*Iidem* AND SULLIVAN, M.—(1960) *Blood*, **15**, 360.
STEVENS, L. AND STOCKEN, L. A.—(1963) *Biochem. J.*, **87**, 12.
STROMINGER, J. L., MAXWELL, E. S., AXELROD, J. AND KALCKAR, H. M.—(1957) *J. biol. Chem.*, **224**, 79.
TAKEMORI, A. E. AND GLOWACKI, G. A.—(1962) *Biochem. Pharmacol.*, **11**, 867.
TRAMS, E. G., INSCOE, J. K. AND RESNIK, R. A.—(1961) *J. nat. Cancer Inst.*, **26**, 959.
WU, R. AND WILSON, D. W.—(1956) *J. biol. Chem.*, **223**, 195.